# Molecular Symphony of Mitophagy: Ubiquitin‐Specific Protease‐30 as a Maestro for Precision Management of Neurodegenerative Diseases

**DOI:** 10.1111/cns.70192

**Published:** 2025-01-22

**Authors:** Ankit Siwach, Harit Patel, Amit Khairnar, Pathik Parekh

**Affiliations:** ^1^ Department of Pharmacology and Toxicology National Institute of Pharmaceutical Education and Research (NIPER) Ahmedabad Gujarat India; ^2^ School of Pharmaceutical Sciences Jaipur National University Jaipur Rajasthan India; ^3^ Department of Physiology, Faculty of Medicine Masaryk University Brno Czech Republic; ^4^ International Clinical Research Center (ICRC) St. Anne's University Hospital Brno Czech Republic; ^5^ International Clinical Research Center (ICRC), Faculty of Medicine Masaryk University Brno Czech Republic; ^6^ Drug Design & Development Section, Translational Gerontology Branch, Intramural Research Program, National Institute on Aging National Institutes of Health Baltimore USA

**Keywords:** Alzheimer's disease, deubiquitinating enzymes, mitophagy, Parkinson's disease, ubiquitin‐specific protease, USP13 inhibitors, USP14 inhibitors, USP30 inhibitors

## Abstract

**Introduction:**

Mitochondrial dysfunction stands as a pivotal feature in neurodegenerative disorders, spurring the quest for targeted therapeutic interventions. This review examines Ubiquitin‐Specific Protease 30 (USP30) as a master regulator of mitophagy with therapeutic promise in Alzheimer's disease (AD) and Parkinson's disease (PD). USP30's orchestration of mitophagy pathways, encompassing PINK1‐dependent and PINK1‐independent mechanisms, forms the crux of this exploration.

**Method:**

A systematic literature search was conducted in PubMed, Scopus, and Web of Science, selecting studies that investigated USP's function, inhibitor design, or therapeutic efficacy in AD and PD. Inclusion criteria encompassed mechanistic and preclinical/clinical data, while irrelevant or duplicate references were excluded. Extracted findings were synthesized narratively.

**Results:**

USP30 modulates interactions with translocase of outer mitochondrial membrane (TOM) 20, mitochondrial E3 ubiquitin protein ligase 1 (MUL1), and Parkin, thus harmonizing mitochondrial quality control. Emerging novel USP30 inhibitors, racemic phenylalanine derivatives, N‐cyano pyrrolidine, and notably, benzosulphonamide class compounds, restore mitophagy, and reduce neurodegenerative phenotypes across diverse models with minimal off‐target effects. Modulation of other USPs also influences neurodegenerative disease pathways, offering additional therapeutic avenues.

**Conclusions:**

In highlighting the nuanced regulation of mitophagy by USP30, this work heralds a shift toward more precise and effective treatments, paving the way for a new era in the clinical management of neurodegenerative disorders.

## Graphical abstract

Novel Ubiquitin‐Specific Protease‐30 (USP30) inhibitors target a key deubiquitinating enzyme, USP30, promoting the ubiquitination and subsequent clearance of dysfunctional mitochondria through the mitophagy pathway. This process offers therapeutic benefits in neurodegenerative diseases by enhancing neuronal cell health.

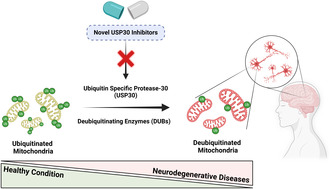

**Abbreviations**
6‐OHDA: 6‐Hydroxydoppamine
AD: Alzheimer's disease
AMBRA1: autophagy and Beclin1 Regulator 1
APP: amyloid precursor protein
ATGs: autophagy‐related genes
Aβ: amyloid‐β
BACE1: β‐site of amyloid precursor protein‐cleaving enzyme
Bak: BCL2 antagonist/killer
Bax: BCL2 associated X
BCL2: B‐cell lymphoma 2
BNIP3: BCL2‐Interacting Protein‐3
BNIP3L: BNIP3 and its homologoue NIX
c‐Abl: Cellular homolog of the Abelson murine leukemia virus oncogene
CMA: Chaperone‐Mediated Autophagy
CRISPR/Cas9: Clustered Regularly Interspaced Short Palindromic Repeats/CRISPR‐associated protein 9
Drp1: dynamin‐related protein 1
DUB: deubiquitinating enzyme
FBXO7: F‐box only protein 7
FBXW7: WD40 domain protein 7
FUNDC1: FUN14 Domain Containing 1
HIF‐1α: hypoxia‐inducible factor‐1α
HOPS: Homotypic Fusion and Protein Sorting
HSP: heat shock cognate protein
IMM: inner mitochondrial membrane
iPSCs: induced pluripotent stem cells
KO: knockout
LAMP2A: lysosome‐associated membrane protein 2A
LBs: Lewy Bodies
LPS: lipopolysaccharide
LRRK2: Leucine‐Rich Repeat Kinase 2
MEFs: mouse embryonic fibroblasts
Mfn: mitofusin
MPP+: 1‐methyl‐4‐phenylpyridinium
mPTP: mitochondrial permeability transition pore
MPTP: 1‐methyl‐4‐phenyl‐1,2,3,6‐tetrahydropyridine
mTOR: mammalian target of rapamycin
NBR1: BRCA1 gene 1 protein
NDP52: nuclear dot protein 52 kDa
Nrf2: nuclear factor erythroid 2‐related factor 2
OMM: outer mitochondrial membrane
OPA1: Optic Atrophy 1
OPTN: optineurin
p62/SQSTM1: Sequestosome 1
PARK2/Parkin: Parkinson protein 2
PARL: Presenilin‐associated rhomboid‐like protein
PD: Parkinson's disease
PINK1: PTEN‐induced putative kinase 1
PLEKHM1: Pleckstrin Homology Domain‐Containing Family M Member 1
PS1: Presenilin‐1
Rab7: Ras‐related protein Rab‐7A
ROCK2: Rho kinase 2
ROS: reactive oxygen species
SAR: structural activity relationship
siRNAs: short‐interfering RNAs
SNc: substantia nigra pars compacta
SREBF1: sterol regulatory element binding transcription factor 1
TAX1BP1: Tax1 (human T‐cell leukemia virus type‐I) binding protein 1
TH: tyrosine hydroxylase
TOM: translocase of the outer mitochondrial membrane
UCHL5: ubiquitin C‐terminal hydrolase L5
USP30: ubiquitin‐specific protease 30

## 1 Introduction

Over the past decade, the upward trajectory of global life expectancy has begun to plateau, posing new challenges [1]. This is particularly concerning, as aging heightens vulnerability to a myriad of chronic diseases, significantly increasing the socioeconomic burdens, especially in developing countries [2, 3]. This demographic shift has intensified the need to better understand the biological processes that maintain cellular health and those that lead to disease [4]. Among these processes, autophagy plays a crucial role in cellular housekeeping and stress response [5]. This conserved catabolic mechanism degrades and recycles cellular debris, including misfolded proteins, such as α‐Synuclein [6, 7, 8], tau [9], and huntingtin [10, 11, 12], as well as redundant and/or damaged organelles like mitochondria, the endoplasmic reticulum (ER), and peroxisomes [13]. Autophagy sustains cell viability under adverse conditions by recycling essential nutrients and proteins from dying cells to benefit viable ones. Diminished or loss of autophagic activity contributes to aging and plays a key role in the onset of neurodegenerative diseases such as Alzheimer's disease (AD), Parkinson's disease (PD), and Huntington's disease (HD), and related neurodegenerative pathologies [14].

Autophagy encompasses three distinct types based on the mechanism of cargo delivery to lysosomes: Chaperone‐Mediated Autophagy (CMA), microautophagy, and Macroautophagy [5]. Among them, macroautophagy (hereafter referred to as autophagy) is the most extensively studied and involves the formation of “autophagosome” that fuses with a lysosome to create an autophagolysosome, leading to the degradation of its contents [15]. Autophagy can target specific organelles, such as mitochondria, peroxisomes, and ribosomes, a process known as mitophagy, pexophagy, and ribophagy, respectively [16]. Among these, mitophagy has garnered significant attention for its intricate role in cellular health and its potential implication in the neurodegenerative pathologies.

This review aims to delve into the complexities of mitophagy, exploring its signaling pathways. Emphasis will be placed on the repercussions of dysfunctional mitophagy in the neurodegenerative disorders, such as AD and PD, reinstating the imperative need for mitophagy enhancers. A critical focus will be on the emerging role of Ubiquitin‐Specific Proteases (USPs), particularly USP30, a deubiquitinating enzyme (DUB), as a novel target for therapeutic intervention. By examining the potential of USP30 inhibitors to restore mitophagy, this review will highlight innovative strategies for combating the intricate nexus between mitochondrial dysfunction and neurodegenerative disorders.

## 2 Mitophagy: A Peculiar Autophagy of Superfluous Mitochondria

Mitochondria are vital for cellular health due to their role in energy production, but they are also prone to damage from reactive oxygen species (ROS) generated during oxidative phosphorylation [17]. When mitochondria become dysfunctional, they perpetuate oxidative damage, leading to cellular dysfunction and cell death [18]. Consequently, the selective and efficient removal of damaged mitochondria, termed mitophagy, is critical for preserving cellular viability. This process, first identified over a century ago, underscores the importance of removing damaged organelles to prevent disease progression [19]. Mitophagy operates through two main pathways in mammals, both of which are contingent on ubiquitin signaling: the PTEN‐induced putative kinase 1 (PINK1)/Parkinson protein 2 (PARK2/Parkin)‐mediated pathway and the receptor‐mediated, PINK1/Parkin‐independent pathway [20]. Despite extensive research into these mechanisms, the specific implications of their differential activation across various tissue types and their overall impact on mitochondrial clearance efficacy remain to be fully elucidated. Delving into these nuances is essential to comprehensively understand mitophagy's role in cellular health and its potential therapeutic applications across neurodegenerative diseases.

## 3 The Multifaceted Signaling Mechanisms Governing Mitophagy

### 3.1 The PINK1/Parkin Pathway: A Cornerstone of Mitophagy

The mitophagy is intricately regulated by PINK1, a mitochondrial serine/threonine kinase, and Parkin, a cytoplasmic E3‐ubiquitin ligase, both of which are pivotal in facilitating the selective degradation of dysfunctional mitochondria [21, 22]. Their involvement is particularly noted in the context of neurodegenerative diseases, such as AD and PD [23, 24]. In PD, mutations in either protein can disrupt mitochondrial quality control, accumulating damaged mitochondria [25, 26]. PINK1 operates as a mitochondrial stress sensor, and its functionality hinges on mitochondrial membrane potential (MMP). PINK1 and Parkin regulates the turnover of outer mitochondrial membrane (OMM) proteins, orchestrates the biogenesis of mitochondria‐derived vesicles, and facilitates the degradation of mitochondria through mitophagy [27]. This balance is achieved through the targeted ubiquitination of mitochondrial proteins, such as mitofusins (Mfn1 and Mfn2) and the mitochondrial fusion protein, dynamin‐related protein 1 (Drp1), thereby segregating damaged mitochondria from their healthy counterparts [28].

**FIGURE 1:**
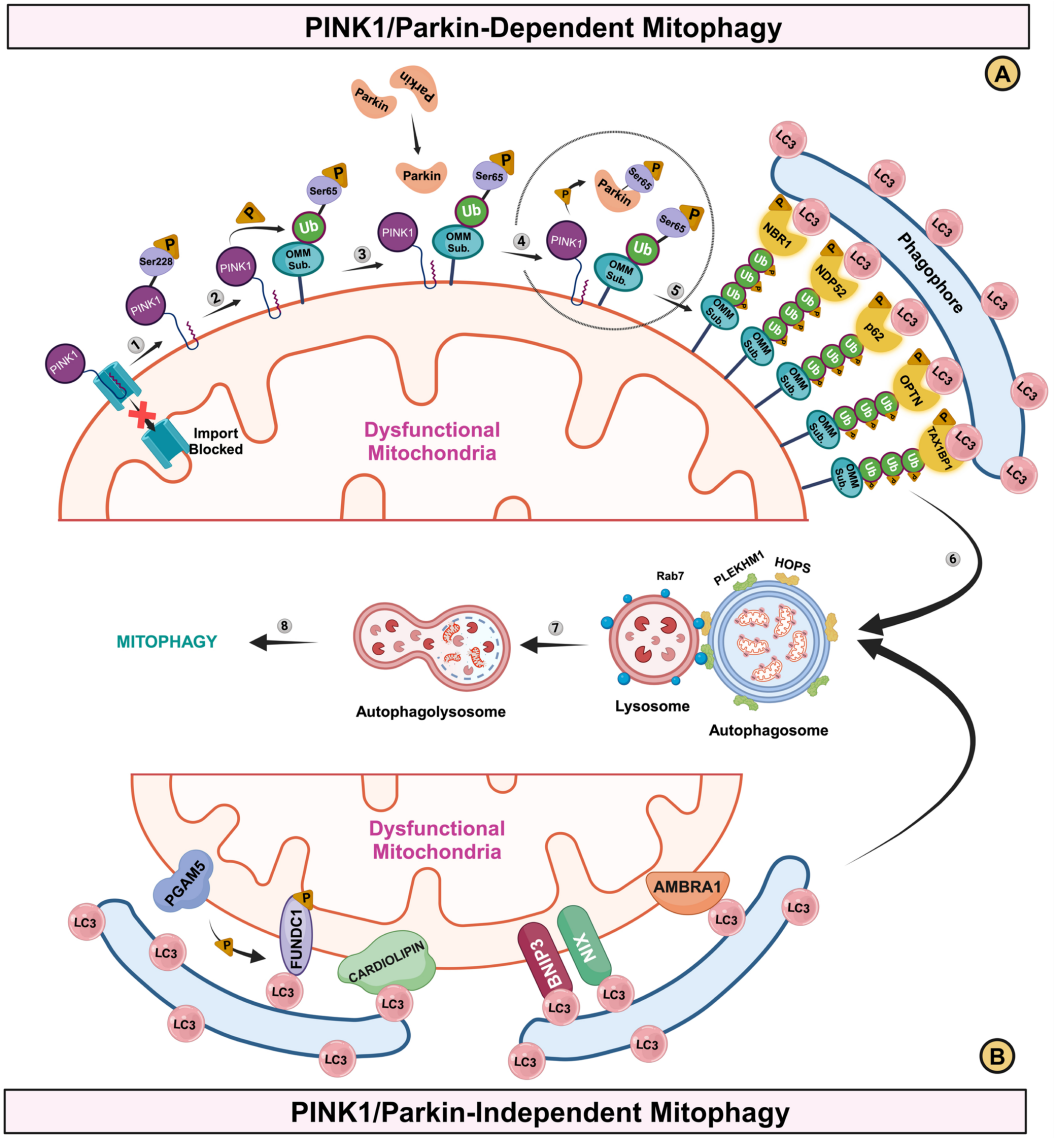
Schematic overview of mitophagy mechanisms. (A) PINK1/Parkin‐dependent mitophagy: (1) Upon mitochondrial depolarization, PINK1 is stabilized at the OMM and activated by auto‐phosphorylation at Ser228. (2) Activated PINK1 phosphorylates ubiquitin at Ser65 position, constitutively attached to OMM proteins. (3) Phosphorylated ubiquitin recruits Parkin to the OMM. (4) Parkin is activated by phosphorylation at Ser65 by PINK1 and by binding to phosphorylated ubiquitin (also phosphorylated at Ser65 by PINK1). (5) Activated Parkin ubiquitinates various OMM substrates (TOM20 and MUL1), which are phosphorylated by PINK1, enhancing further ubiquitination and Parkin recruitment in a positive feedback loop. These ubiquitin chains act as “eat me signals,” leading to the recruitment of autophagy receptors (NBR1, NDP52, p62, OPTN, and TAXBP1), which then bridge the ubiquitinated mitochondria to the LC3‐positive autophagosome. Phosphorylation of autophagy receptors by TBK1 increases interaction between LC3 and ubiquitin. (6) The autophagosome engulfs the ubiquitinated mitochondria. (7) Autophagosome fuses with the lysosome via PLEKHM1 and HOPS complex (autophagosome side) and Rab7 (lysosomal side), allowing for the degradation of the autophagosome contents by the lysosome hydrolases. (8) These series of events culminate in effective mitophagy. (B) PINK1/Parkin‐Independent mitophagy: Triggered by mitochondrial depolarization, OMM mitophagy receptors, including FUNDC1, Cardiolipin, BNIP3, NIX, and AMBRA1 mediate binding to LC3 through their LIR domains and promote the mitophagy.

Under physiological conditions, PINK1 is imported into the inner mitochondrial membrane (IMM), where it undergoes proteolytic cleavage by the presenilin‐associated rhomboid‐like protein (PARL), an IMM protease [29]. This cleavage facilitates PINK1's subsequent degradation via the N‐degron pathway [30], a process crucial for preventing its accumulation [31]. However, when mitochondria are damaged, PINK1 accumulates on the OMM, where, it interacts with the translocase of the outer membrane (TOM) complex (Figure 1A). Recent advancements have provided detailed insights into the structural organization of PINK1 stabilized on the OMM. These studies reveal that N terminal region of PINK1 traverses the pore of the TOM complex, engaging directly with TOM20, and simultaneously establishes contract with the components of the translocase of the inner mitochondrial membrane (TIM), specifically TIM17 and TIM23 [32, 33]. This results in the assembly of a PINK1‐TOM‐TIM23 supercomplex, which is critical for PINK1‐mediated mitophagy signaling [32, 33]. In addition, TIM23 prevents OMA1 (Zinc metalloendopeptidase)‐induced degradation of PINK1 in damaged mitochondria, promoting its accumulation [32]. Furthermore, PINK1 initiates a pivotal phosphorylation cascade upon its activation, where PINK1 phosphorylates both ubiquitin molecules and Parkin itself at Ser65 residues [34]. This phosphorylation event activates Parkin and enhances its binding affinity to phosphorylated‐ubiquitin, facilitating a series of conformational changes that further potentiate Parkin's enzymatic activity [35, 36, 37, 38, 39]. Once activated, Parkin ubiquitinates key proteins on the OMM to the depolarized mitochondria. This tagging can occur through the elongation of pre‐existing ubiquitin chains on OMM proteins or through the de novo ubiquitination of OMM substrates, a testament to the versatility of Parkin's enzymatic functions [40].

Autophagy receptors such as Next to BRCA1 gene 1 protein (NBR1) [41], Nuclear dot protein 52 kDa (NDP52) [42, 43], Optineurin (OPTN) [44], Sequestosome 1 (p62/SQSTM1) [45], and Tax1 (human T‐cell leukemia virus type‐I) binding protein 1 (TAX1BP1) [42, 46] play a key role in recognizing these ubiquitinated mitochondria and forming autophagosomes around them. The final stage of mitophagy involves the fusion of autophagosomes with lysosomes, a process coordinated by specialized proteins such as Pleckstrin Homology Domain‐Containing Family M Member 1 (PLEKHM1) and the Homotypic Fusion and Protein Sorting (HOPS) complex on the autophagosome side and Ras‐related protein Rab‐7A (Rab7) on the lysosome side, culminating in the degradation of the targeted mitochondria [47].

Recent advancements have identified the DUBs, particularly USPs, as novel pharmacological targets for neurodegenerative diseases, including AD, PD, and HD. Among all USPs, USP30, located on the OMM, antagonizes the mitophagy by removing ubiquitin chains added by Parkin, thus highlighting the dynamic interplay between ubiquitination and deubiquitination in mitochondrial quality control [48]. This insight into USP30's role enriches our understanding of mitochondrial dynamics and underscores the therapeutic potential of targeting DUBs to modulate mitophagy.

### 3.2 Beyond PINK1/Parkin: Other Avenues of Mitophagy

While the PINK1/Parkin pathway is central to mitochondrial quality control, research has uncovered PINK1/Parkin‐independent mitophagy pathways [49] (Figure 1B). Studies using PINK1‐deficient mice [50] and *Drosophila melanogaster* deficient in either PINK1 or Parkin [51] have shown that basal mitophagy can occur without PINK1 or Parkin, indicating the existence of other regulatory mechanisms. In these alternative pathways, various mitophagy receptors play crucial roles in targeting damaged mitochondria to degradation from the proximity of dysfunctional neurons, a phenomenon observed in many neurodegenerative diseases [52]. These receptors, located on the OMM, are structurally equipped with a transmembrane domain and an LC3‐interacting region (LIR) motif, allowing them to bind autophagosomes. Key examples include B‐cell lymphoma 2 (BCL2)‐Interacting Protein‐3 (BNIP3), NIX, FUN14 Domain Containing 1 (FUNDC1), Autophagy and Beclin1 Regulator 1 (AMBRA1), and cardiolipin [49].

#### 3.2.1 BNIP3 and NIX: Pioneers in Alternative Mitophagy Pathways

BNIP3 and its homolog NIX (BNIP3L), initially recognized for their role in apoptosis, have emerged as critical regulators of mitophagy [53, 54] (Figure 1B). Despite their milder apoptotic influence, these proteins are essential in inducing mitophagy in response to mitochondrial dysfunction [55, 56]. Under stress condition, such as hypoxia, BNIP3 expression is upregulated by hypoxia‐inducible factor‐1α (HIF‐1α), facilitating the removal of damaged mitochondria to preserve cellular energy homeostasis. The hypoxia‐induced homodimerization of BNIP3, a critical step for its mitophagic activity, promotes its incorporation into the OMM, highlighting its regulatory role in mitochondrial turnover [57]. BNIP3‐mediated mitophagy is also triggered by lipotoxicity, where excessive lipid accumulation promotes mitochondrial depolarization [58], and calcium‐dependent activation of Drp1 [59]. The breadth of triggers for BNIP3‐mediated mitophagy highlights its versatile role in cellular defense against a range of mitochondrial stressors. In the context of neurodegenerative diseases, BNIP3 downregulation has been associated with mitochondrial dysfunction in dopaminergic neurons [60]. Furthermore, downregulation of BNIP3 homodimer has been observed in a murine model of PD having heterozygous mutations in the Glucocerebrosidase (GBA) gene, a common genetic risk factor for PD [61]. Thus, abovementioned studies suggest that BNIP3 may act as a parallel molecular pathway that critically regulates mitophagy independent of PINK1 and Parkin. However, the exact interplay between BNIP3/NIX and the PINK1/Parkin pathway remains unclear and warrants further investigation.

#### 3.2.2 FUNDC1: Hypoxia‐Induced Mitophagy Receptor

FUNDC1, an integral OMM protein, orchestrates hypoxia‐induced mitophagy, illustrating the cell's adaptive response to oxygen deprivation [62]. Unlike the PINK1/Parkin pathway, FUNDC1 directly interacts with LC3 on autophagosomes (Figure 1B), a process finely tuned by its phosphorylation status [63].

Under normoxic conditions, the mitochondrial E3‐ubiquitin ligase MARCH5 targets FUNDC1 for proteasomal degradation through ubiquitylation at Lys119 to prevent uncontrolled mitophagy [62]. However, under hypoxia or stress from treatments like carbonyl cyanide‐p‐trifluoro‐methoxy‐phenylhydrazone (FCCP), mitochondrially localized phosphatase, PGAM5 dephosphorylates FUNDC1 at Ser13, promoting its interaction with LC3 and activating mitophagy [62]. FUNDC1's activity is further regulated by BCL2L1/Bcl‐xl, which inhibits its dephosphorylation and downregulates mitophagy in the absence of stress [64]. Beyond its role in mitophagy, FUNDC1 interacts with proteins involved in mitochondrial dynamics, such as Drp1 and Optic Atrophy 1 (OPA1) [65]. This interaction contributes to the regulation of mitochondrial morphology and the overall integrity and function of the mitochondrial network, indicating FUNDC1's pivotal role in maintaining cellular energy homeostasis under stress. The ability of FUNDC1 to modulate mitophagy in response to cellular stress through a delicate balance of phosphorylation states emphasizes its significance in mitochondrial quality control, offering insights into how disruptions in this balance may contribute to the pathology of neurodegenerative diseases.

#### 3.2.3 AMBRA1: A Versatile Regulator of Mitophagy

AMBRA1, initially recognized for its role in autophagy, has emerged as a key regulator of mitophagy. Activated under hypoxic conditions, AMBRA1 translocate inside mitochondria and interacts with LC3 through its LIR motif, inducing mitophagy in either Parkin‐independent or Parkin‐dependent manners [65, 66, 67] (Figure 1B). This flexibility is particularly relevant in neurodegenerative disease models, where AMBRA1 has been shown to protect against neurotoxins like 6‐Hydroxydopamine (6‐OHDA) and rotenone‐induced oxidative stress and neuronal death [66]. In addition, AMBRA1 overexpression at OMM promoted Parkin‐ and p62‐independent but LC3‐dependent mitophagy in cells derived from PD patients lacking PINK1 [67]. AMBRA1 underscores the complex regulatory networks that maintain cellular and mitochondrial health by bridging autophagy and mitophagy. Its role in mitigating stress and disease states offers a promising avenue for research into treatments for neurodegenerative diseases, where mitochondrial dysfunction is a hallmark.

#### 3.2.4 Cardiolipin: A Novel Modulator of Mitophagy

Cardiolipin, a phospholipid localized to the IMM, plays a pivotal role in the mitophagy and apoptosis. Under normal conditions, it supports mitochondrial function and energy production [68]. During low and mild stress, cardiolipin facilitates localization of Drp1 to damaged mitochondria, assisting the mitochondrial fission [69]. The daughter mitochondrion with lower MMP undergoes mitophagy, guided by binding of LC3 to cardiolipin on the OMM [69] (Figure 1B). The daughter mitochondria with intact MMP may fuse with a healthy mitochondrion for repair [69]. During apoptotic or high stress, cardiolipin relocates to the OMM [70] and becomes more prone to the attack by ROS, because of its high unsaturated fatty acid content [71]. This translocation serves as a signal for identifying damaged mitochondria, leading to the recruitment of pro‐apoptotic protein, Bax, opening of the mitochondrial permeability transition pore (mPTP), release of cytochrome c from mitochondria and promotes apoptosis [69]. The role of cardiolipin extends to neurodegenerative diseases, notably PD, where its interaction with pathological proteins like α‐Synuclein fibrils exacerbates mitochondrial damage and worsens PD pathology [72]. This interaction highlights cardiolipin's role in promoting mitochondrial clearance and potentially contributing to disease pathology.

## 4 Perturbed Mitophagy and Its Implications in the Neurodegenerative Pathologies

Mitochondrial dysfunction is pivotal in a spectrum of neurodegenerative diseases, highlighting the critical significance of mitophagy in preserving cellular health. As aging progresses, a notable reduction in mitophagy leads to the accumulation of damaged mitochondria [73]. This phenomenon is particularly salient in neurodegenerative disorders, such as AD and PD, where heightened oxidative stress and misfolded proteins significantly contribute to the disease progression.

### 4.1 Alzheimer's Disease (AD)

AD, the preeminent neurodegenerative disorder, is characterized by a profound cognitive decline rooted in energy deficiency and synaptic inhibition [74]. Neuropathologically, AD entails the aggregation of extracellular amyloid‐β (Aβ) protein and intracellular neurofibrillary tangles containing hyperphosphorylated tau protein [75]. These pathogenic hallmarks arise from inadequate mitochondrial function, producing ROS and subsequent denaturation of proteins, lipids, and nucleic acids [76]. Furthermore, elevated ROS promotes the accumulation of pathological form of Aβ protein and phosphorylated‐tau protein which ultimately forms Aβ‐plaques and neurofibrillary tangles, inducing mitochondrial impairments, thus creating a vicious cycle [77, 78]. The exposure of synaptic terminals to Aβ protein further exacerbates mitochondrial dysfunction and oxidative stress [79]. Recent in vivo studies exploring novel pharmacotherapy and lifestyle‐modifying interventions for AD have unveiled the potential benefits of mitigating mitochondria‐specific oxidative stress, enhancing mitochondrial biogenesis, and restoring mitophagy through strategies like fasting and physical exercise [80, 81]. Notably, mitophagy enhancers such as Urolithin‐A and Actinonin have demonstrated significant improvements in learning and memory deficits in the amyloid precursor protein (APP) mouse model of AD [82, 83]. Furthermore, stimulation of mitophagy in neurons derived from AD patients reduces hyperphosphorylation of tau protein [84], indicating the crucial role of mitophagy impairments in AD pathogenesis and signifies the need for innovative mitophagy enhancers in AD treatment.

### 4.2 Parkinson's Disease (PD)

PD, the second most prevalent neurodegenerative disorder, is characterized by progressive degeneration of nigrostriatal dopaminergic neurons [85]. This further produces a dopamine deficiency in the striatum, manifesting as the characteristic motor impairments observed in PD patients [85]. Central to the neuropathological landscape of PD are neurodegeneration, neuroinflammation, oxidative stress, and notably, mitochondrial dysfunction, which plays a pivotal role in the onset of PD pathology [86]. Exposure to the neurotoxins like 1‐methyl‐4‐phenyl‐1,2,3,6‐tetrahydropyridine (MPTP) and rotenone promotes mitochondrial dysfunction through the inhibition of mitochondrial complex‐1 [87, 88, 89]. This further exemplifies the vulnerability of mitochondria in PD and accentuates the critical need for an efficient mitophagy process in counteracting mitochondrial damage.

The process of mitophagy is regulated by crucial modulators, F‐box containing proteins, including F‐box only protein 7 (FBXO7), sterol regulatory element binding transcription factor 1 (SREBF1), and WD40 domain protein 7 (FBXW7), intricately linked to the PINK1/Parkin pathway's efficiency in PD [90]. While SREBF1 takes center stage as a risk factor for sporadic PD [91], genes such as PARK2, PARK6, and PARK15, encoding Parkin, PINK1, and FBXO7 respectively, claim their roles in the autosomal recessive form of PD, intricately involved in PINK1/Parkin‐mediated mitophagy [92]. Beyond PINK1 and Parkin, other virtuoso genes like DJ‐1 (PARK7), α‐Synuclein (SNCA), and Leucine‐Rich Repeat Kinase 2 (LRRK2) contribute to the complex regulation of mitochondrial functions and mitophagy, indicating the multifaceted nature of PD pathology [92]. DJ‐1, a protein encoded by the PARK7 gene, plays a protective role against oxidative stress and supports mitochondrial function and the mutations underlying PD's recessive manifestation [93]. The oxidation event at the Cys106 position of DJ‐1 downgrades the expression of tyrosine hydroxylase (TH), a pivotal enzyme governing dopamine synthesis [94]. Simultaneously, this oxidation triggers an elevation in the levels of nuclear factor erythroid 2‐related factor 2 (Nrf2), a potent orchestrator of the antioxidant stress response [95, 96]. The deletion of DJ‐1 resulted in a notable upregulation of oxidative stress, disturbance in mitochondrial fission, and alterations in mitophagy, providing additional evidence for the protective roles of DJ‐1 in maintaining mitochondrial functions [93, 97]. The iconic α‐Synuclein, encoded by PARK1, etches its presence in aggregated form within Lewy Bodies (LBs), further influencing mitochondrial health through its intricate connection with the TOM20 [98, 99]. Moreover, α‐Synuclein's interaction with cardiolipin has been spotlighted as crucial mechanism that facilitates the recruitment of LC3 to damaged mitochondria, thereby promoting mitophagy [72]. Conversely, a pathogenic form of α‐Synuclein plays a discordant role, altering mitophagy through its interaction with cardiolipin [100].

This complex regulatory network of mitochondrial maintenance, influenced by genetic factors and protein interactions, highlights the significance of altered mitophagy in the pathogenesis of neurodegenerative diseases. The evolving understanding of mitophagy dysregulation deepens our insight into development of pathologies and opens new therapeutic pathways for tackling neurodegenerative diseases. Targeting these pathways with pharmacological agents to enhance or restore mitophagy offers promising therapeutic potential for neurodegenerative disorders, such as AD and PD [101, 102, 103].

## 5 Therapeutic Effects of Mitophagy Enhancers in Neurodegenerative Disorders

Existing shreds of evidence strongly laud the occurrence of mitochondrial dysfunction in several neurodegenerative disorders [104]. Consequently, enhancing the clearance of dysfunctional mitochondria by promoting/restoring mitophagy seems to be a disease‐modifying avenue. Over the last decade, our comprehension of mitophagy pathways has expanded significantly (as described in Section 3), leading to the identification of potential therapeutic targets and pharmacological agents acting on those targets capable of activating mitophagy. So far, several natural compounds and synthetic molecules have been demonstrated to upregulate mitophagy and subsequently exert neuroprotection in multiple neurodegenerative diseases including AD and PD [105, 106] (Table 1).

**TABLE 1:** Conventional mitophagy enhancers and their protective effects in neurodegenerative disorders.

Sr. No.	Compound (Dose and route of administration)	Target/s	Effects observed	In vivo and/or In vitro model	References
1.	Urolithin A (In vitro: 2.5, 5 and 10 μM, 2 h) (In vivo: 20 mg/kg/day, 7 days, i.p.)	Mitophagy activation	Promoted mitophagy and restored mitochondrial dysfunction Reduced activation of astrocytes and microglia and counteracted dopaminergic neuron loss Restored MPTP‐induced behavioral deficit	LPS‐exposed murine microglia cells (BV2) (1 μg/mL) MPTP mouse model (15 mg/kg/every 2 h, four times a day)	[83]
2.	Nilotinib (In vivo: 25 mg/kg/day, 14 days, i.p.)	c‐Abl	Reduced MPTP‐induced c‐Abl activation and levels of Parkin substrate (PARIS) Prevented MPTP‐induced loss of dopaminergic neurons and behavioral deficits	MPTP‐induced mouse model (20 mg/kg/2‐h × 4‐times/day, i.p.)	[82]
3.	Nilotinib (In vivo; 30 μL/day, 3‐weeks, i.p.)	c‐Abl	Increased endogenous Parkin level owing to increased autophagic changes Increased ubiquitination and proteasomal recycling of Parkin, reduced insoluble Parkin, enhanced Parkin‐Beclin1 interaction and ultimately promoted Aβ_1–42_ clearance	Bilateral injection of Aβ_1–42_ into CA1 of the hippocampus of 1‐year‐old C57BL/6 or Parkin KO mice	[107]
4.	Compound 3 (In vitro; 10 μM for 30 h)	Miro 1	Eliminates Miro1 from depolarized mitochondria Rescued locomotor deficits and neurodegeneration in PD and enhanced mitophagy	Fibroblasts derived from the skin of PD patients PD flies	[108]
5.	Rapamycin (In vivo; 1 mg/kg/day × 2 months, i.p.)	Parkin	Increased Parkin‐mediated mitophagy and promoted fusion of mitophagosome and lysosome in the hippocampus Rapamycin enhanced learning and memory, synaptic plasticity, and the expression of synapse‐related proteins Attenuated cytochrome C‐mediated apoptosis, decreased oxidative stress and restored mitochondrial function	APP/PS1 mouse model of AD	[109]
6.	VP07 (In vitro; 25 μM)	PINK1/ Parkin	Restored mitophagy impairments induced by Paraquat and 6‐OHDA	Paraquat and 6‐OHDA‐treated SH‐SY5Y cells	[110]
7.	T0466 and T0467	NA	Promoted Parkin's mitochondrial translocation in dopaminergic neurons and promoted mitophagy Reduced unfolded mitochondrial protein levels Mitigated the PINK1 inactivation‐mediated larval locomotion defects, mitochondrial morphological defects and reduced ATP production	Dopaminergic neurons Muscle‐specific PINK1 KO in *Drosophila*	[111]
8.	Pramipexole (In vitro; 1, 10, 100 μM × 24 h) (In vivo: 1 mg/kg/day, 7‐days, i.p.)	BNIP3‐mediated mitophagy	Promoted the clearance of dysfunctional mitochondria and attenuated neuronal injury and apoptosis Activated BNIP3‐mediated mitophagy via downregulation of miR‐96	MPP^+^‐treated SH‐SY5Y and SK‐N‐SH cells (2.5 mM MPP^+^ for 24 h) MPTP‐treated mouse model (30 mg/kg/day for 5‐consecutive days, i.p.)	[112]
9.	SR3677 (In vitro; 0.5 μM × 2 h) (In vivo; 1 mM × 7‐days; for mitoQC assay) (In vivo; 1 mM × 4‐days; for longevity and climbing assay)	ROCK2	Promoted the activity of PINK1/Parkin pathway by recruiting Hexokinase 2, a positive regulator of Parkin, to mitochondria Increased targeting of mitochondria to lysosomes and removed damaged mitochondria Improved locomotor ability of Paraquat‐treated flies	SH‐SY5Y cells (CCCP treatment: 10 μM × 40 min) Paraquat‐based drosophila model of PD (Paraquat: 1 mM × 7‐days; for mitoQC assay) (Paraquat: 10 mM × 4‐days; for longevity and climbing assay)	[113]
10.	BC1464 (In vitro; 5 μg/mL × 24 h)	FBXO7	Abrogated FBXO7‐PINK1 interaction and thereby increased cellular PINK1 levels and attenuated mitochondrial damage	MPP^+^‐treated SH‐SY5Y neuroblastoma cells (400 μM MPP^+^ for 24 h) Mouse primary cortical neurons PD patient‐derived fibroblasts	[114]
11.	GSK3357679A (In vivo: 15 mg/kg/every 12‐h × 4 times)	LRRK2 inhibition	Rescued the LRRK2‐induced mitophagy defects	LRRK2 G2019S mutant mice	[115]

Urolithin A, a gut‐microbiota‐derived metabolite of pomegranate juice, has emerged as a compelling mitophagy enhancer [83]. Urolithin A restores mitochondrial function and activate mitophagy, thereby mitigating neuroinflammation and neurodegeneration in lipopolysaccharide (LPS)‐treated microglial cells and MPTP‐induced mouse models of PD [83]. Another noteworthy player is c‐Abl (cellular homolog of the Abelson murine leukemia virus oncogene). This endogenous tyrosine kinase inhibits mitophagy via modulation of Parkin by phosphorylating Tyr143 residue [116]. Indeed, increased levels of phosphorylated c‐Abl have been observed in the striatum and substantia nigra of PD patient's brains, demonstrating its pathogenic role in PD pathology [116]. Nilotinib, functioning as a c‐Abl inhibitor, exhibited its protective effects in wild‐type (WT) and Parkin knockout (KO) mouse models subjected to bilateral injection of Aβ_1–42_ into the hippocampus [82]. Nilotinib reduced not only amyloid protein load through the induction of mitophagy, facilitated by elevated Parkin levels but also counteracted behavioral impairments and degeneration of nigrostriatal dopaminergic neurons in mice subjected to acute MPTP treatment [82].

In recent advancements, Miro1, an OMM GTPase crucial for mitochondrial trafficking, has emerged as a critical player linked with impaired mitochondrial clearance in familial and sporadic PD [117]. The significance of Miro1 in initiating mitophagy has been emphasized by a recent study characterizing compound‐3 as a mitophagy enhancer, primarily acting through Miro1 inhibition [108]. Remarkably, compound‐3 displayed the ability to lower Miro1 levels post‐mitochondrial depolarization, providing neuroprotection in patient‐derived PD fibroblasts and Drosophila models, marking a significant advancement in therapeutic strategies [108].

Rapamycin, a renowned autophagy/mitophagy inducer, operates by inhibiting the mammalian target of rapamycin (mTOR) [118]. Notably, rapamycin alleviated AD‐like phenotypes and synaptic plasticity deficits in the hippocampus of the APP/ Presenilin‐1 (PS1) mouse model [109]. These protective effects were concomitant with the activation of Parkin‐mediated mitophagy [109]. This finding aligns with the therapeutic potential of autophagy/mitophagy modulation in neurodegeneration.

Recent phenotypic screening led by Maestro et al. has identified two novel synthetic mitophagy enhancers, VP07 and JAR1.39. These compounds restore mitophagy alterations induced by neurotoxins, such as paraquat and 6‐OHDA in dopaminergic neuroblastoma (SH‐SY5Y) cells, providing a new direction for therapeutic development [110].

In a groundbreaking revelation, a cell‐based high‐throughput screening uncovered the potential of two small molecules, T0466 and T0467, poenchmarking a highly selective USP30 inhibitor for enhatent inducers of mitophagy [111]. These compounds promote Parkin's translocation from the IMM to OMM within midbrain dopaminergic neurons [111]. Furthermore, their therapeutic effects extend to mitigating aggregation of damaged mitochondria in dopaminergic neurons of Drosophila, a consequence of reduced PINK1 activity [111]. This compelling evidence suggests the promising therapeutic application of T0466 and T0467 in neurodegenerative disorders characterized by mitochondrial dysfunction [111].

Adding to the intrigue, pramipexole, a widely employed dopamine D_2_‐receptor agonist traditionally used in PD treatment, has shown remarkable efficacy in promoting BNIP3‐mediated mitophagy [112]. This effect was observed in both MPP^+^‐treated SH‐SY5Y and SK‐N‐SH cells, as well as in the MPTP‐treated mouse model of PD [112]. These findings shed light on the potential multifaceted applications of pramipexole and reinstate the intricate interplay between dopamine signaling and mitophagy, unveiling novel dimensions in pursuing of targeted therapies for neurodegenerative diseases.

In 2020, Moskal and colleagues discovered SR3677, a small‐molecule inhibitor of Rho kinase 2 (ROCK2), with a unique mechanism of mitophagy induction. Unlike many enhancers that act on mitochondrial damage or apoptosis, SR3677 promotes mitophagy by increasing the recruitment of Parkin to damaged mitochondria [113]. This compound activates the PINK1/Parkin pathway by recruiting Hexokinase 2, a positive regulator of Parkin, to damaged mitochondria in SH‐SY5Y cells [113]. Additionally, SR3677 improves the targeting of superfluous mitochondria to lysosomes and removes damaged mitochondria in a Paraquat‐induced drosophila model of PD [113]. This novel approach challenges conventional methods, offering a promising avenue for mitophagy modulation through ROCK2 inhibition.

The studies outlined above robustly endorse the protective effects of both natural and novel synthetic mitophagy enhancers, showcasing their potential to promote and restore mitophagy within the landscape of neurodegenerative pathologies. While the predominant focus of existing research has centered on the PINK1/Parkin‐dependent pathway, there arises an imperative to delve into novel therapeutics targeting alternative pathways. This exploration holds the promise of yielding interventions that might surpass the efficacy of conventional approaches. In this context, DUBs, pivotal regulators of mitophagy, have garnered heightened attention in recent investigations. Recognizing their significant role in orchestrating the delicate balance of mitophagy, studies probing DUBs stand poised to unveil novel insights and potential therapeutic avenues. These endeavors promise to extend beyond the current paradigm, offering fresh perspectives for more efficacious interventions in neurodegenerative disorders.

## 6 Mitophagy Regulation by Deubiquitinating Enzymes (DUBs): Impact on Neurodegenerative Diseases

DUBs stand as master regulators within the cellular milieu, playing a critical role in the orchestration of mitophagy. Their influence extends to the very core of mitophagy machinery, where they exercise control over Parkin's activity through the direct deubiquitination of either Parkin or its substrates, thus intricately fine‐tuning the mitophagy response [119]. The human genome encodes approximately 80 active DUBs, each contributing uniquely to cellular homeostasis and proteostasis [120, 121, 122, 123]. Notable among these are enzymes such as USP8 [124, 125, 126], USP13 [127, 128], USP15 [121], USP30 [120, 129], and USP35 [123], which have been closely associated with the regulation of mitophagy. These DUBs display varied impacts on mitophagy, either promoting or inhibiting the process, thereby influencing mitochondrial turnover and overall cellular health. Beyond their roles in mitophagy, DUBs are involved in several critical cellular processes. They are responsible for processing inactive ubiquitin precursors into active ubiquitin molecules necessary for tagging proteins destined for degradation [130]. DUBs also proofread ubiquitin‐protein conjugates by removing incorrect ubiquitin attachments, ensuring specificity in protein degradation pathways [130]. They recycle ubiquitin from substrates before degradation, maintaining the ubiquitin pool within the cell [130]. Additionally, DUBs preserve the functionality of the 26S proteasome by removing inhibitory ubiquitin chains, which is crucial for degrading misfolded or damaged proteins [130]. Dysregulation of these processes can lead to proteostasis imbalance, contributing to the accumulation of toxic protein aggregates, a hallmark of many neurodegenerative disorders.

Among the myriads of DUBs, USP30 is particularly intriguing due to its distinct localization and function. Unlike its counterparts, USP30 is constitutively associated with the OMM, positioning its catalytic domain toward the cytoplasm, the very domain housing Parkin [131]. This unique placement allows USP30 to modulate mitochondrial ubiquitination and deubiquitination, making it a key player in the regulation of mitophagy. By counteracting Parkin‐mediated ubiquitination of mitochondrial proteins, USP30 inhibits the clearance of damaged mitochondria, potentially contributing to mitochondrial dysfunction. The significance of DUBs in neurodegeneration extends beyond USP30. For instance, USP13 influences the stability of mitochondrial proteins and has been implicated in the regulation of mitophagy and proteasomal degradation of pathogenic proteins, such as α‐Synuclein and tau [127, 132]. USP14 modulates proteasome activity and exhibits a dual role, potentially exerting cytoprotective effects in some contexts [133], while contributing to mitochondrial dysfunction in others [134]. Other DUBs, such as USP8 and USP15, participate in protein degradation and mitochondrial dynamics, with alterations in their activity linked to neurodegenerative processes [121, 135, 136].

Understanding the roles of DUBs in mitophagy provides valuable insights into the molecular mechanisms underlying neurodegenerative diseases. Aberrant DUB activity impairs removal of dysfunctional mitochondria, increases oxidative stress, and neuronal cell death. Consequently, DUBs represent promising therapeutic targets. Modulating their activity offers the potential to correct mitophagy defects, reduce mitochondrial dysfunction, and mitigate neuronal loss associated with neurodegenerative conditions. The following section will delve deeper into the specific actions and implications of key DUBs such as USP30, USP8, USP13, USP14, USP15, and USP33. We will explore their unique capabilities, broader impact on mitochondrial quality control and cellular health, and impact on the neurodegenerative diseases, highlighting opportunities for therapeutic intervention.

### 6.1 USP30 and Neurodegenerative Diseases

**FIGURE 2:**
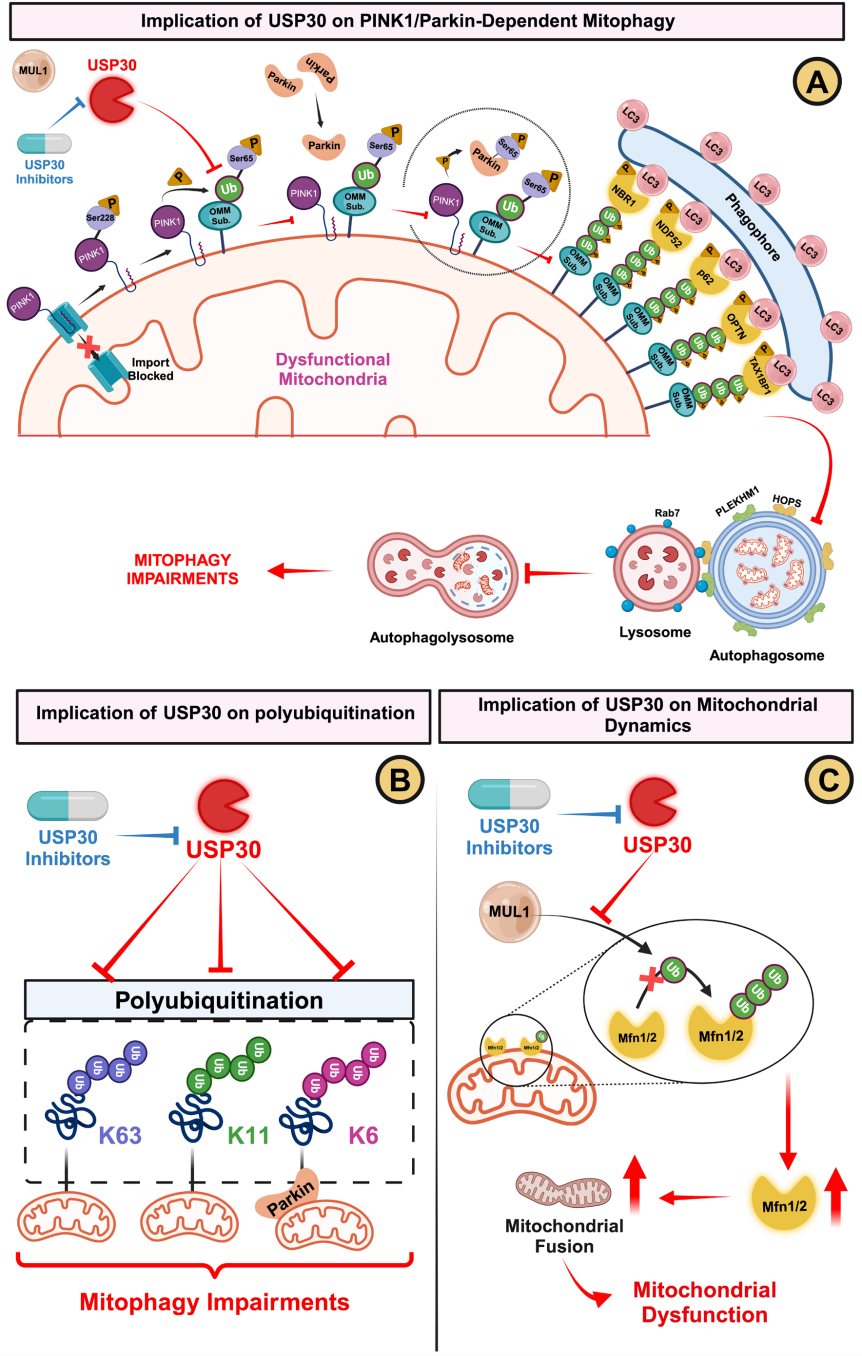
The Role of Ubiquitin‐Specific Protease (USP)‐30 in Mitophagy Impairments and Effects of USP30 Inhibitors (A) PINK1/Parkin‐dependent mitophagy: USP30 acts to deubiquitinate OMM proteins such as TOM20. This action impedes Parkin recruitment and activation at the OMM, leading to a failure to recruit autophagy receptors necessary for tagging damaged mitochondria. Consequently, this inhibits the fusion of autophagosomes with lysosomes, ultimately blocking the mitophagy process. USP30 inhibitors rescue the mitophagy impairments by inhibiting the USP30. (B) Polyubiquitination: USP30 selectively removes non‐canonical (K6 and K11‐linked) and canonical (K63‐linked) ubiquitin chains from damaged mitochondria and Parkin. This activity hinders the proper functioning of mitophagy, creating impairments in the clearance of dysfunctional mitochondria. USP30 inhibitors rescues the mitophagy impairments by inhibiting the USP30‐induced deubiquitination of K6‐, K11‐, and K63‐linked ubiquitin chains from damaged mitochondria and Parkin. (C) Mitochondrial dynamics: By deubiquitinating Mfn1 and Mfn2, USP30 causes an elevation in these proteins, promoting mitochondrial fusion. This alteration in mitochondrial dynamics can further impede effective mitophagy, leading to an accumulation of dysfunctional mitochondria. USP30 inhibitors promote the ubiquitination of Mfn1/2, prevent the accumulation of damaged mitochondria, and restore mitophagy. HOPS, Homotypic Fusion and Protein Sorting; LC3, microtubule‐associated proteins 1A/1B light chain 3A; Mfn, Mitofusin; NBR1, next to BRCA1 gene 1 protein; NDP52, nuclear dot protein 52 kDa; OMM, outer mitochondrial membrane; OPTN, optineurin; p62, Sequestosome 1 (SQSTM1); PLEKHM1, Pleckstrin Homology Domain‐Containing Family M Member 1; Rab7, Ras‐related protein Rab‐7As; TAX1BP1, Tax1 (human T‐cell leukemia virus type‐I) binding protein 1;TOM20, translocase of outer mitochondrial membrane 20; Ub, ubiquitin.

USP30, a novel mitochondrial DUB, emerges as a key player in orchestrating mitophagy dynamics, as it fine‐tunes the removal of damaged mitochondria by deubiquitinating OMM adaptor proteins, especially TOM20 [120, 137] (Figure 2A). This action places USP30 at the heart of mitophagy regulation, inhibiting the PINK1‐ and Parkin‐mediated pathways crucial for mitochondrial quality control [120, 137] (Figure 2A). Notably, catalytically inactive mutant USP30 fails to impede mitophagy in vivo, suggesting the regulatory significance of USP30 in this process [120, 138]. USP30 exhibits a nuanced specificity for ubiquitin chains, proficiently cleaving both non‐canonical (Lys6 and Lys11) and canonical (Lys63) chains [138] (Figure 2B). These ubiquitin chains, preferentially added to damaged mitochondria, facilitate the selective removal of superfluous mitochondria. Intriguingly, USP30 displays diminished activity toward phosphorylated polyubiquitin chains, emphasizing its preferential engagement with Lys6‐linked ubiquitin chains, prevalent in Parkin substrates, further justifying the specificity of USP30 for PINK1/Parkin pathway [139] (Figure 2B). USP30's impact is not only limited to PINK1/Parkin pathways, it also plays a role in PINK1‐independent mitophagy, adding layers to its function within mitochondrial dynamics [140].

Beyond TOM20, USP30 interacts with mitochondrial E3 ubiquitin‐protein ligase 1 (MUL1), a shared substrate with Parkin that influences mitochondrial fusion and dynamics by regulating ubiquitin‐dependent degradation of mitochondrial fusion proteins, Mfn1 and Mfn2 [141] (Figure 2C). MUL1's protective effects have been demonstrated in disorders involving mitochondrial defects, including PD, as MUL1 counteracts deleterious effects owing to the loss of PINK1 or Parkin in fruit flies [141]. Crucially, MUL1 stands out as the predominantly ubiquitylated protein among the shared substrates of USP30 and Parkin. Consequently, the deubiquitination of MUL1 through innovative USP30 inhibitors emerges could mitigate excessive mitochondrial fusion and reinstate mitophagy in the context of neurodegenerative diseases. Beyond mitophagy, USP30 mediates Bax/BCL2 Antagonist/Killer (Bak)‐dependent apoptosis, hinting at its involvement in programmed cell death pathways [137]. Intriguingly, a distinct pool of USP30 resides in peroxisomes alongside mitochondria, regulating the basal level of pexophagy in a PINK1‐independent manner [140]. As mitochondria and peroxisomes intricately influence each other and contribute to cellular ROS, USP30's control over their turnover aligns with its potential implications in the pathogenesis of neurodegenerative diseases. The intricate functions of USP30 in mitochondrial quality control position it as a significant factor in the pathogenesis of neurodegenerative diseases characterized by mitochondrial dysfunction and oxidative stress. Its role as a negative regulator of mitophagy has garnered increasing attention, especially in the context of PD and AD.

In PD, mitochondrial dysfunction is closely linked with the genetic and environmental factors contributing to the pathology [142]. Studies have shown that overexpression of USP30 inhibits Parkin‐dependent mitophagy in neuroblastoma and human cells, leading to impaired clearance of damaged mitochondria [120]. Conversely, inhibition of USP30 restores mitophagy and offers neuroprotective effects [120]. In Parkin‐ or PINK1‐deficient drosophila, USP30 knockdown increases the recruitment of the autophagy receptor SQSTM1/p62 to damaged mitochondria, thereby restoring mitophagy impairments and preserving mitochondrial integrity [120]. Knockdown of USP30, particularly in dopaminergic neurons in vivo, not only conferred significant protection against paraquat‐induced toxicity but also restored dopamine levels and alleviated motor deficits [120]. These findings highlight the therapeutic potential of USP30 inhibition in rescuing mitophagy defects associated with PD, both in vitro and in vivo [120]. Moreover, the safety profile of USP30 inhibition, evidenced by the absence of adverse effects in USP30 KO mice [120], supports the feasibility of this therapeutic approach. Cultured neurons from these KO models show increased mitophagy [120], suggesting that USP30 inhibition could mitigate neurodegenerative pathology without inducing toxicity.

While the direct role of USP30 in AD is less extensively studied, its involvement in mitochondrial quality control positions it as a significant factor in the disease's mitochondrial dysfunction narrative. Given the central role of mitochondria in neuronal health and function and the emerging evidence linking mitochondrial dysfunction with AD pathology, modulating USP30 activity presents a novel strategy for addressing AD's underlying causes. A recent study by Jiang and colleagues supported the role of USP30 overexpression in the development of AD‐specific impairments [143]. As, the findings demonstrated the elevated levels of USP30 in the post‐mortem brains of AD patients [143]. Of interest, *in vivo* experiments showed that microRNA‐137‐5p significantly suppressed USP30 levels and attenuated Aβ_1–42_‐induced neurotoxicity in SH‐SY5Y cells [143]. In addition, it restored cognitive and motor impairments in AD mice through hippocampus‐ and cortex‐specific downregulation of Aβ_1–42_ deposition, tau hyperphosphorylation, and neuronal apoptosis [143]. These findings suggest that modulating USP30 activity could be a novel strategy for addressing mitochondrial dysfunction and neuronal loss in AD.

While the role of USP30 in HD remains unclear, a study by Escarcega et al. offers valuable insights [144]. They explored the impact of sphingosine kinase (SPHK) isoforms, SPHK1 and SPHK2, key enzymes in the synthesis of sphingosine‐1‐phosphate (S1P), on neuronal protein ubiquitination [144]. Overexpression of SPHK2 in cultured primary striatal neurons led to an increase in ubiquitin substrates [144]. Conversely, pharmacological inhibition of SPHK2 resulted in the downregulation of several ubiquitination‐related proteins, including E3 ubiquitin ligases (HUWE1 and TRIP12), the E2 ubiquitin‐conjugating enzyme (UBE2Z), and ubiquitin‐specific proteases (USP15 and USP30) [144]. Elevated levels of SPHK2 and HUWE1 were observed in the striatum of HD mouse model [144]. These findings suggest that dysregulation of the ubiquitination pathway, potentially involving USP30, contributes to HD pathology, highlighting the need for further research into USP30's role in this disease.

As research continues to elucidate the complex mechanisms underlying neurodegenerative diseases, the role of USP30 as a negative regulator of mitophagy stands out as a promising target for therapeutic intervention. Future studies aimed at understanding USP30's precise mechanisms of action and the development of specific, safe, and effective USP30 inhibitors could revolutionize the treatment landscape for neurodegenerative conditions characterized by mitochondrial dysfunction.

### 6.2 USP8 and Neurodegenerative Diseases

USP8 has emerged as a critical regulator within the cellular environment, with its actions extending from modulation of protein aggregates to fine‐tuning of mitochondrial dynamics. Influencing neurodegenerative disease pathologies, mainly through its interaction with α‐Synuclein and mitochondrial quality control mechanisms, positions USP8 as a critical target for therapeutic intervention.

**FIGURE 3:**
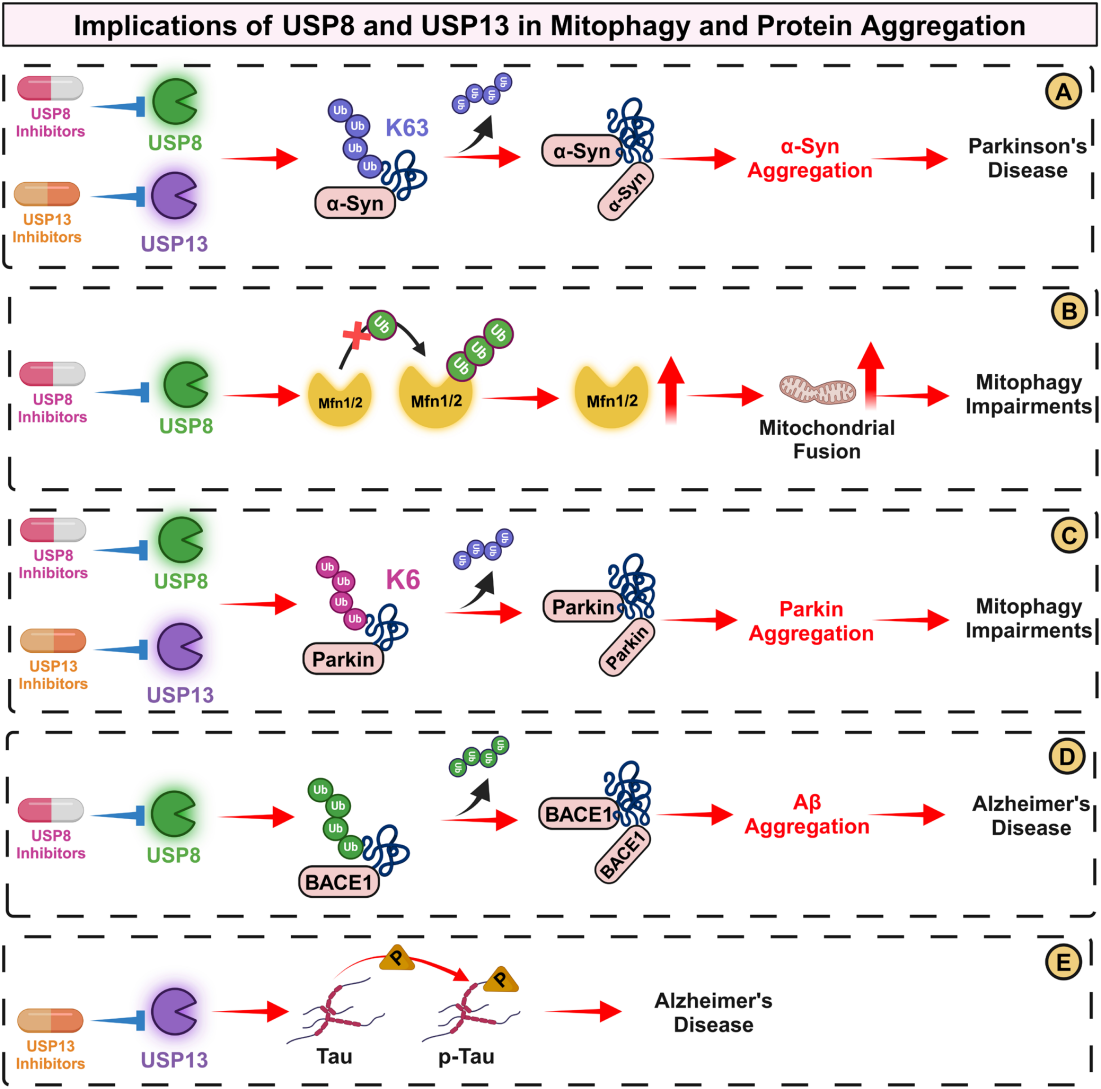
Impact of Ubiquitin‐Specific Protease (USP)‐8 and USP13 on Mitophagy and Protein Aggregation (A) α‐Synuclein Aggregation: USP8 and USP13 deubiquitinates K63‐linked ubiquitin chains on α‐Synuclein, promoting its aggregation. This process leads to the development of Parkinson's disease (PD) pathology. USP8 and USP13 inhibitors restore the ubiquitination of K63‐linked ubiquitin chains on α‐Synuclein, thus mitigating its aggregation. (B) Mitochondrial Dynamics: USP8 deubiquitinate Mfn1 and Mfn2. The increase in these proteins disrupts mitochondrial dynamics, contributing to mitophagy impairments. USP8 inhibitors rescue mitophagy impairments by promoting the ubiquitination of Mfn1/2. (C) Parkin and Mitophagy: USP8 and USP13 remove K6‐linked ubiquitin chains from Parkin on the outer mitochondrial membrane (OMM). This alteration leads to Parkin accumulation on the OMM and hinders efficient mitophagy. USP8 and USP13 inhibitors restore the ubiquitination of K6‐linked ubiquitin chains from Parkin, thus promoting mitophagy. (D) USP8 and BACE1: USP8 acts to deubiquitinate BACE1, leading to its accumulation. Elevated BACE1 levels enhance the production of amyloid‐beta (Aβ) protein, which aggregates and is implicated in the pathogenesis of Alzheimer's disease (AD). USP8 inhibitors restore the ubiquitination of BACE1, preventing the aggregation of Aβ protein. (E) Tau Phosphorylation by USP13: USP13 is involved in the phosphorylation of tau protein, a process that contributes to the neurofibrillary tangles characteristic of AD. USP13 inhibitors prevent the phosphorylation of tau protein, thus mitigating the formation of neurofibrillary tangles. BACE1, Beta‐Site Amyloid Precursor Protein (APP)‐Cleaving Enzyme 1; Mfn, mitofusin.

USP8 directly impacts the pathogenesis of PD by modulating α‐Synuclein, a protein whose aggregation is a hallmark pathological feature of PD [135]. USP8 fosters α‐Synuclein aggregation by deubiquitinating K63‐linked ubiquitin chains, a modification that stabilizes α‐Synuclein in a prone‐to‐aggregate form [135] (Figure 3A). Furthermore, endogenous USP8 knockdown in drosophila eyes reduces α‐Synuclein aggregation and mitigates associated toxicity [135]. This protective effect extends to human cellular models, where USP8 knockdown promotes lysosomal degradation of α‐Synuclein, and to midbrain dopaminergic neurons, where it safeguards against α‐Synuclein‐induced dysfunction [135], illuminating its critical role in PD pathology.

Beyond its involvement in α‐Synuclein dynamics, USP8's influence extends to mitochondrial quality control, highlighting its importance in cellular health. USP8 regulates Mfn expression, a critical factor in mitochondrial fusion and integrity [136] (Figure 3B). In PINK1 and Parkin‐deficient drosophila, both genetic and pharmacological inhibition of USP8 acted as a restoration catalyst, enhancing Mfn levels [136]. This restoration, in turn, has led to improved mitochondrial function, alleviated locomotor deficits, and the protected dopaminergic neurons, highlighting the detrimental effects of dysregulated USP8 activity on mitochondrial health [136]. USP8 also dampened mitophagy by removing K6‐linked ubiquitin chains on Parkin [145] (Figure 3C). Although crucial for effective recruitment and subsequent removal of dysfunctional mitochondria, this step poses an intricate regulatory challenge [145].

The scope of USP8's impact spans beyond PD, as evidenced by a study from Yeates et al., shedding light on its involvement in AD [146]. Where USP8's deubiquitinating action on the β‐site of amyloid precursor protein‐cleaving enzyme (BACE1), a pivotal enzyme in the production of neurotoxic peptide, Aβ, has been elucidated [146] (Figure 3D). USP8‐mediated deubiquitination of BACE1 exacerbates Aβ accumulation, a key event in AD pathogenesis [146] (Figure 3D). Conversely, USP8 downregulation curtailed BACE1 levels, mitigating Aβ production [146]. These intriguing findings hint at the potential therapeutic promise of pharmacological agents, such as USP8 inhibitors that may promote BACE1 degradation and provide protection in AD and PD.

The intricate roles of USP8 in modulating both α‐Synuclein aggregation and mitochondrial quality control across PD and AD highlight its potential as a therapeutic target. The development of selective USP8 inhibitors could offer a dual benefit: mitigating protein aggregation and enhancing mitochondrial function. Such pharmacological agents hold promise for addressing the complex interplay of pathogenic processes in neurodegenerative diseases, offering a targeted approach to treatment that could slow disease progression and alleviate symptoms. The evolving narrative surrounding USP8's contributions to neurodegenerative disease pathology demonstrates the enzyme's potential as a therapeutic target. By unraveling the mechanisms by which USP8 modulates essential pathogenic proteins and mitochondrial dynamics, the research paves the way for novel interventions to restore cellular homeostasis in neurodegenerative disorders.

### 6.3 USP13 and Neurodegenerative Diseases

USP13, nestled within the broad superfamily of cysteine‐dependent proteases, intricately partakes in the deubiquitination process, reversing the ubiquitin‐dependent degradation of protein substrates, notably α‐Synuclein and tau [147] (Figure 3). Its function in neurodegeneration has garnered attention, particularly given the observed upregulation of USP13 in *post‐mortem* analyses of PD patient's midbrains, indicating a significant link to PD pathology [127]. In preclinical models of α‐Synucleinopathies, USP13 assumes a dual regulatory role, independently influencing the ubiquitination of both Parkin and α‐Synuclein, critical players in PD pathogenesis [127] (Figure 3A,C). Pertinently, shRNA‐induced USP13 knockdown amplifies α‐Synuclein ubiquitination, facilitating its clearance independently of Parkin. This intervention results in notable improvements in locomotor function and preservation of nigral dopaminergic neurons from α‐Synuclein‐induced neurotoxicity in the transgenic models of α‐Synucleinopathies [127].

The tale of USP13 extends beyond PD, making an entrance into the realms of AD, as USP13 was found to be upregulated in the brains of patients with AD [132]. A pivotal study in tau‐overexpressing mouse primary cortical neurons unravels USP13's influence on phosphorylated‐tau levels, demonstrating that USP13 knockdown reduces tau phosphorylation via enhanced proteasomal activity [132] (Figure 3E). This regulatory mechanism points to USP13's potential in mitigating tau pathology, a hallmark of AD and related tauopathies. Transcending in vivo boundaries, USP13 knockdown in transgenic APP mice unfolds a dual impact, diminishing Aβ levels and boosting the ubiquitination‐mediated clearance of phosphorylated‐tau protein [132]. This dual effect propels USP13 into the spotlight as a potential therapeutic target, hinting at the prospect of USP13 inhibitors as novel interventions in the intricate tapestry of neurodegenerative diseases. The elucidation of USP13's regulatory mechanisms in neurodegenerative diseases highlights its potential as a target for therapeutic intervention. The development of USP13 inhibitors could offer a novel strategy for addressing the complex molecular pathologies of PD and AD. By enhancing the ubiquitination and clearance of pathogenic proteins such as α‐Synuclein and phosphorylated‐tau, USP13 inhibition could provide a multifaceted approach to ameliorating disease progression and symptoms.

### 6.4 Other USPs in the Pathogenesis of Neurodegenerative Diseases

The involvement of other DUBs such as USP14, USP15, and USP33 further complicates the landscape of neurodegenerative diseases. These enzymes extend the regulatory network governing autophagy and mitophagy, essential for cellular homeostasis and mitochondrial quality control.

USP14, prominently featured in the aging process, exhibit upregulated expression in the cerebellum and substantia nigra pars compacta (SNc) of adult animals [148]. This upregulation is further enhanced in response to neurotoxic insults, such as rotenone [148]. Inhibition of USP14 ameliorates mitochondrial dysfunction and locomotor deficits, particularly those associated with PINK1/Parkin mutations, suggesting a vital role of USP14 in modulating SNc‐specific mitophagy and neurodegeneration [134, 148]. Furthermore, USP14‐mediated impairments in mitophagy are dependent on mitochondrial dynamics proteins DRP1 and Mfn2 and can occur independently of PINK1 and Parkin [134]. In contrast to above findings, USP14 has also been found to exert cytoprotective effects and reduce cell aggregates in mutant huntingtin (mtHtt)‐expressing cells [133]. It also counteracts ER stress‐mediated IRE1α (serine/threonine‐protein kinase/endoribonuclease inositol‐requiring enzyme 1α) activation in mtHtt cells [133]. Taken together, these observations suggest that USP14 plays a complex, dual role in neurodegenerative diseases, acting as both a potential therapeutic target and a protector of neuronal health, depending on the disease context. Its ability to regulate both mitophagy and proteasomal degradation highlights the need for a nuanced approach in therapeutic development. Future research should focus on delineating the precise mechanisms by which USP14 exerts its effects in different neurodegenerative conditions to harness its therapeutic potential effectively.

USP15, abundantly present in the brain, emerges as a pivotal antagonist to Parkin‐mediated mitophagy [121]. Knockdown studies of USP15 have shown restoration of Parkin levels and mitigation of mitophagy defects in fibroblasts derived from PD patients with PARK2 mutations [121]. These findings position USP15 inhibition as a promising therapeutic approach, especially in PD cases with diminished Parkin activity [121]. Drosophila models further support this notion, where the knockdown of USP15's homolog reinstates mitochondrial health and alleviates behavioral impairments [121].

USP33, a recent entrant in the neurodegenerative narrative, directly interacts with Parkin, unlike the other USPs to inhibit the mitophagy process [149]. USP33 preferentially removes the K6‐, K11‐, K48‐, and K63‐linked ubiquitin chains from Parkin, inhibiting mitophagy [149]. Notably, USP33 knockdown restores Parkin's stability and translocation to dysfunctional mitochondria, protecting the neuroblastoma cells against MPTP‐induced neuronal death [149]. The strategic inhibition of USP33 emerges as a novel therapeutic strategy, further expanding the potential avenues for managing neurodegenerative diseases.

While USP14, USP15, and USP33 offer new insights into the regulation of mitochondrial quality control and neurodegeneration, the prominent role of USP30 remains undiminished. USP30's involvement in regulating PINK1/Parkin‐dependent mitophagy, polyubiquitination, mitochondrial dynamics and the modulation of neurodegenerative disease progression highlights its significance as a therapeutic target. The exploration of USP30 inhibitors holds considerable promise for developing targeted interventions in the multifaceted realm of neurodegenerative disorders.

## 7 Comparative Analysis of Ubiquitin‐Specific Proteases (USPs), Their Collective Impact, and Possible Interplay in Neurodegenerative Diseases

As discussed previously (Section 6), various USPs such as USP8, USP13, USP14, USP15, USP30, and USP33 are vital regulators of the ubiquitin‐proteasome system (UPS), influencing key cellular processes such as protein degradation, mitochondrial dynamics, and mitophagy in neurodegenerative diseases. Although direct studies evaluating the interplay among these USPs in neurodegenerative contexts are currently lacking, a comparative analysis of their individual functions may provide valuable insights into their collective impact on disease progression.

All these USPs commonly regulate ubiquitination processes but differ in substrate specificity, cellular localization, and mechanisms of action. For instance, USP30 is uniquely localized to the OMM and directly regulates mitophagy by deubiquitinating mitochondrial substrates like TOM20, Mfn1, and Mfn2 [120, 137, 141]. By counteracting Parkin‐mediated ubiquitination, USP30 inhibits the clearance of damaged mitochondria, contributing to mitochondrial dysfunction [120]. In contrast, USP15 and USP33 inhibit mitophagy by directly interacting with Parkin [121, 149]. It removes ubiquitin chains from Parkin itself, impairing its stability and translocation to dysfunctional mitochondria, thereby impeding the mitophagy process. USP14, predominantly associated with the proteasome, regulates overall protein turnover by deubiquitinating substrates destined for degradation. It exhibits a dual role in neurodegeneration, potentially exacerbating mitochondrial dysfunction in PD by impairing mitophagy [134], while exerting cytoprotective effects in HD by reducing mtHtt protein aggregates and counteracting ER stress [133]. USP8 fosters α‐Synuclein aggregation by deubiquitinating K63‐linked ubiquitin chains on α‐Synuclein, contributing to PD pathology [135]. It also regulates mitofusin expression, affecting mitochondrial fusion and integrity, which are critical for neuronal function [136]. USP13 modulates degradation of Parkin, α‐Synuclein, and tau‐proteins and mitochondrial dynamics [127, 132]. Its inhibition promotes the clearance of α‐Synuclein and improves mitochondrial health. Collectively, USP30, USP33, and USP15 impede the removal of damaged mitochondria, increase oxidative stress, and neuronal death, while USP8, USP13, and USP14 influence the aggregation and degradation of pathogenic proteins such as α‐Synuclein, tau, and mtHtt. By stabilizing these proteins or affecting their degradation pathways, they exacerbate protein aggregation, contributing to neuronal toxicity and disease progression.

While each USPs operates through distinct mechanisms, their activities may overlap or compensate for one another due to their involvement in similar pathways. For example, both USP30 and USP33 negatively regulate mitophagy, albeit through different targets, mitochondrial substrates versus Parkin itself. This suggests that inhibiting one USP may not fully restore mitophagy if another USP with a similar function remains active. Additionally, the inhibition or loss of one USP could potentially be offset by the upregulation or increased activity of another USP with overlapping functions. Understanding these potential compensatory mechanisms is crucial for developing effective therapeutic strategies. The lack of direct studies evaluating the interplay among these USPs highlights a significant gap in our understanding of their collective impact on neurodegeneration. Although direct evidence of interplay among various USPs in neurodegenerative diseases is currently lacking, the comparative analysis of their functions underscores their collective significance in disease progression. Recognizing both the similarities and differences among these enzymes is essential for understanding the complex molecular mechanisms underlying neurodegeneration and for developing targeted therapies that address multiple aspects of the pathogenic process.

## 8 Therapeutic Modulation of Ubiquitin‐Specific Proteases (USPs) by Novel USP Inhibitors in the Neurodegenerative Diseases

### 8.1 Novel USP30 Inhibitors

Embarking on a journey through the molecular landscape of neurodegenerative diseases, the scientific community has uncovered a beacon of hope in the form of USP30 inhibitors, a class of compounds poised to redefine therapeutic strategies for conditions characterized by mitochondrial dysfunction. This narrative explores the advent of USP30 inhibitors, their evolution from broad‐spectrum to precision‐targeted agents, and the promise they hold in neurodegeneration.

Initial explorations into the world of USP30 inhibition revealed a spectrum of compounds, ranging from metal‐based inhibitors to sophisticated small molecules. These efforts, while pioneering, often grappled with specificity issues, lacked a precise understanding of their mechanistic actions, and inadvertently affected other DUBs and proteasomal activities. The journey began with broad‐spectrum agents like auranofin and evolved toward compounds with a sharper focus on USP30, illuminating the path toward precise mitochondrial quality control. Numerous researchers and biopharmaceutical companies, including Mission Therapeutics and Forma Therapeutics, are searching for highly specific and potent USP30 inhibitors, targeting USP30 through both covalent and non‐covalent mechanisms. Targeting USP30 with specific inhibitors is a promising avenue to restore mitophagy in conditions characterized by mitochondrial defects, including neurodegenerative diseases.

#### 8.1.1 The Genesis of Selective Inhibition

While nascent, the journey into USP30 inhibition has unveiled compounds with promising capabilities to modulate USP30's expression or enzymatic activity. USP30 inhibitors that work by inhibiting the USP30 expression include antisense oligonucleotides, short‐interfering RNAs (siRNAs), antibodies, peptides, peptibodies, aptamers, small molecules, and metal‐based inhibitors [150]. The versatility of these inhibitors is indicated by their varied administration routes, including oral, intramuscular, intravenous, intra‐arterial, intraperitoneal, or subcutaneous, broadening their therapeutic potential.

Initial forays into metal‐based DUB inhibitors such as auranofin, copper pyrithione, zinc pyrithione, platinum pyrithione, and nickel pyrithione revealed a bias toward proteasomal DUBs like Ubiquitin C‐terminal hydrolase L5 (UCHL5) and USP14, overshadowing their impact on USP30 [150]. Nevertheless, recent advancements highlight a promising shift toward specifically designed USP30 inhibitors.

#### 8.1.2 A New Era of Specificity: Aumdubin and Beyond

Aumdubin emerged as a milestone in this journey, a gold(I)‐containing a derivative of auranofin with a remarkable affinity for USP30 [151]. This gold(I)‐containing agent demonstrated superior DUB‐inhibitory and apoptosis‐inducing effects in lung cancer cells compared to its parent compound, Auranofin [151]. Its capability to induce Bax‐dependent apoptosis and ubiquitination, partially via USP30 inhibition, sets a new benchmark for targeted therapies [151].

**FIGURE 4:**
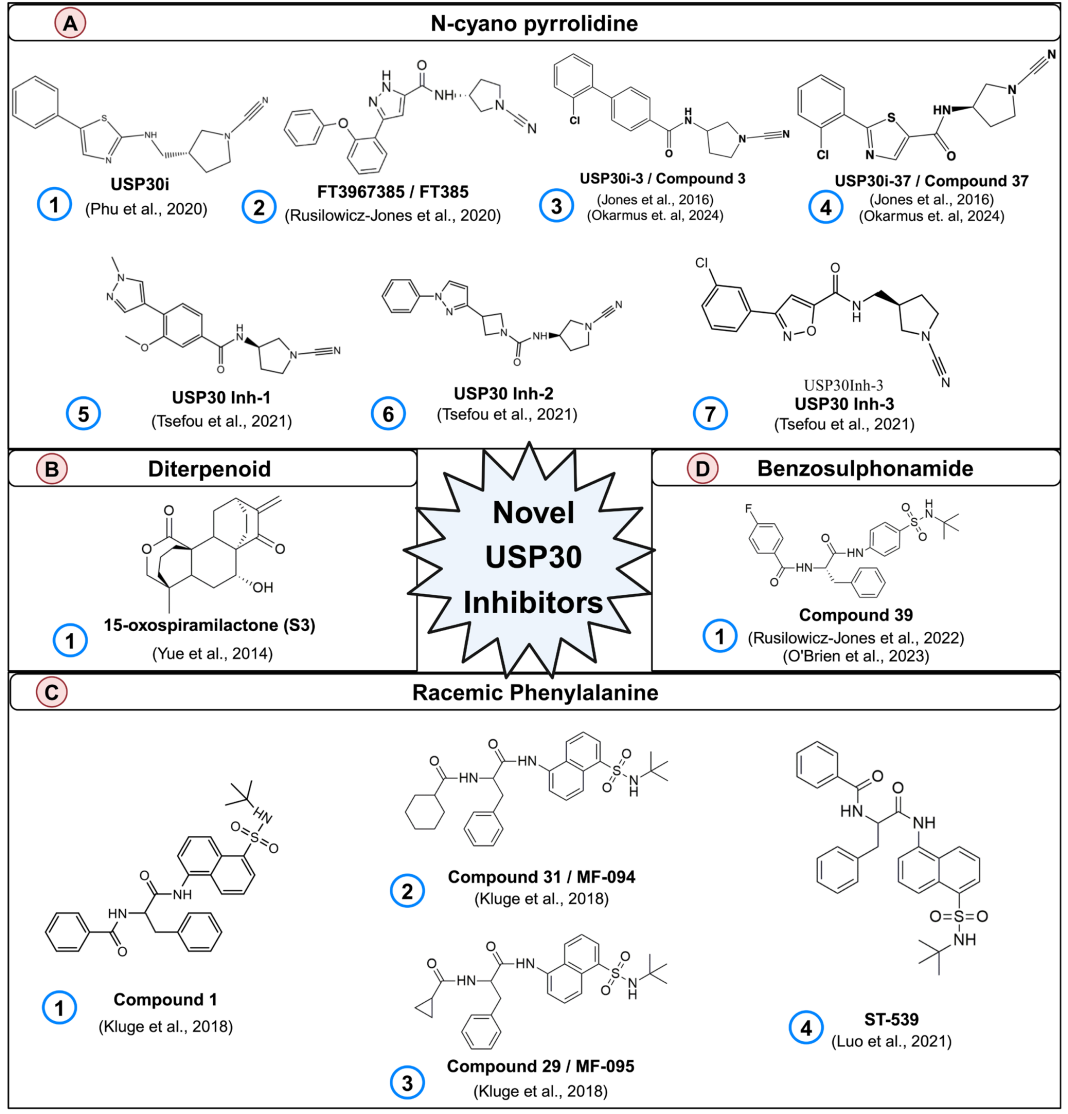
Schematic showing the chemical structures of novel USP30 inhibitors. The novel USP30 inhibitors are separated based on the chemical class: (A) N‐cyano pyrrolidine, (B) Diterpenoid, (C) Racemic phenylalanine derivatives, and (D) Benzosulphonamide.

The discovery of N‐cyano pyrrolidines marked a significant advancement, introducing a class of inhibitors initially inspired by cathepsin‐C inhibitors yet demonstrating activity against USP30 (patents: WO2016156816A1, WO2017009650A1, WO2017163078A1, WO2018060689A1, WO2018060691A1, WO2018060742A1, and WO2018065768A1; Mission Therapeutics) (Figure 4A). These inhibitors showcase activity against UCHL1 and USP30, raising concerns about selectivity and specificity. Forma Therapeutics has disclosed multiple patents detailing compounds with N‐cyano motifs. These patents provide detailed information on the range of USP30 inhibitory activities exhibited by the compounds provided in the patent (11814386). Mission therapeutics also developed a potent USP30 inhibitor, USP30i (MTX115325), a N‐cyano pyrrolidine derivative, which has good oral bioavailability and CNS penetration (WO2021/249909A1). MTX115325 inhibits USP30 with an IC50 if 12–25 nM, depending on the assay and system employed [152]. Notably, either loss of USP30 in USP30 knockout mice or pharmacological inhibition of USP30 by MTX115325 restored mitophagy impairments, reduced phospho‐S129 α‐Synuclein, mitigated α‐Synuclein‐induced nigrostriatal dopaminergic neuron loss, and subsequently restored behavioral deficits [152]. In a recent study, Phu and colleagues evaluated the cellular efficacy of USP30i [129] (Figure 4A: 1). The research indicated that USP30i notably augmented the ubiquitination of TOM20, a recognized USP30 target, achieving an EC50 value of 2.45 μM within BAM1 (mitochondrial depolarizer)‐treated and Parkin‐overexpressing HEK293 cells. Additionally, mass spectrometry analyses were employed to determine the specificity of USP30i toward USP30. The results revealed that while USP30i predominantly targets USP30, it also interacts with a spectrum of other proteins, including several deubiquitinating enzymes (DESI2, ATXN3, UBP4, UBP45, and UBP47). This points to the broader DUB‐engaging potential of USP30i [129].

Furthermore, a modified N‐cyano pyrrolidine derivative, FT3967385/FT385 (Figure 4A: 2), has been developed and characterized for its impact on USP30 [153]. The Ubiquigent DUB profiler screen experiment demonstrated that FT385 exhibited high selectivity for USP30 at lower concentrations (up to 200 nM), but this specificity diminished at higher concentrations [153]. Proteomic analysis using endogenous Parkin‐expressing neuroblastoma SH‐SY5Y cells emphasized FT385's superior selectivity for USP30 compared to conventional compounds in the same class [153]. FT385 replicated the effects of genetic USP30 deletion on mitophagy and significantly enhanced the ubiquitination of TOM20 upon mitochondrial depolarization [153]. It led to TOM20 degradation without affecting PINK1 levels ultimately increasing mitophagy flux in both human retinal pigment epithelial‐1 (RPE‐1) and SH‐SY5Y cells [153]. While the protective effects of FT385 have been demonstrated, caution is warranted due to its potential to enhance the ubiquitination of several proteins beyond TOM20.

While N‐cyano pyrrolidines' specificity and cellular efficacy as USP30 inhibitors have been well reported, their protective effects, particularly in the context of PARK2 mutation carriers, require further exploration. Building on this foundation, Okarmus et al. utilized iPSC technology to create WT and PARK2 KO isogenic human dopaminergic neurons. This innovative approach allowed for a nuanced investigation into how PARK2 deletion influences mitophagy in response to mitochondrial stress and assessed the efficacy of novel compounds—precisely, compounds 3 and 37 from the N‐cyano pyrrolidine class [154] (Figure 4A: 3–4). These compounds were initially described in the patent WO2016/156816 [155]. Findings revealed that these compounds significantly enhance mitophagy and reduce oxidative stress in neurons, irrespective of parkin presence, suggesting their potential to counteract parkin‐independent mitophagy [154]. The above findings hint at a broader applicability of USP30 inhibitors, suggesting their potential therapeutic application in PD and a range of disorders entailing mitochondrial dysfunction. These results demand the need for the development of next‐generation USP30 inhibitors having enhanced specificity and robust selectivity for USP30.

#### 8.1.3 Charting the Course: Innovations and Breakthroughs

The landscape further expanded with USP30Inh‐1, ‐2, and ‐3 synthesis by Tsefou and colleagues [156] (Figure 4A: 5–7). These compounds are based on parent compound structures previously disclosed in various patents (WO/2016/156816 [155] and WO/2017/103614 [157]). Notably, these inhibitors feature a cyano‐amide functional group crucial for inhibiting USP30 by forming a covalent bond with its active site [156]. USP30Inh‐1, 2, and 3 exhibited superior selectivity against more than 40 known DUBs at a concentration of 1 μM. However, selectivity diminished at higher concentrations, with notable off‐target inhibition observed for USP6, USP21, and USP45, possibly attributable to the presence of the cyano‐amide group [156]. Significantly, these inhibitors effectively suppressed USP30‐induced cleavage of ubiquitin‐rhodamine 110 (Ub‐Rho110), an artificial fluorogenic substrate of DUBs, at IC50 values ranging from 15 to 30 nM [156]. Utilizing induced pluripotent stem cells (iPSCs)‐derived dopaminergic neurons, the same study demonstrated that pharmacological inhibition by these inhibitors of USP30 led to an enhanced accumulation of p‐Ser65‐ubiquitin and subsequently promoted mitophagy [156].

In the pursuit of effective USP30 inhibitors, Yue et al. conducted a screening of 300 compounds, leading to the identification of a potent inhibitor named 15‐oxospiramilactone (S3), a diterpenoid derivative (Figure 4B: 1). This compound showcased remarkable capabilities in rescuing mitochondrial architecture and oxidative respiration in both mouse embryonic fibroblasts (MEFs) and human cell lines deficient in Mfn1 and Mfn2 [158]. Notably, S3 exerted its effects by upregulating mitochondrial fusion proteins, Mfn1 and Mfn2, ultimately promoting the fusion of mitochondria [158].

After high‐throughput screening, a racemic phenylalanine derivative was identified, namely compound 1 (Figure 4C: 1), which emerged as a potential USP30 inhibitor with an IC50 value below 1 μM. At concentrations below 10 μM, Compound 1 exhibited higher specificity for USP30 compared to other USPs. Structural activity relationship (SAR) studies derived several compounds from Compound 1, resulting in more potent and specific USP30 inhibitors, including MF‐094 (a potent and selective USP30 inhibitor) (Figure 4C: 2) and MF‐095 (a less potent USP30 inhibitor) [159] (Figure 4C: 3). The impact of MF‐094 is particularly noteworthy, which enhances protein ubiquitination, subsequently promoting mitophagy in terminally differentiated C2C12 myoblasts cells [159]. Furthermore, Luo and colleagues studied the effects of ST‐539, a nex‐generation racemic phenylalanine derivative in HeLa cells (Figure 4C: 4). Findings indicated that ST‐539 induced tissue‐specific mitophagy through USP30 inhibition and exhibited minimal mitochondrial toxicity in HeLa cells, demonstrating good tolerance in mice [160]. Nevertheless, the complete characterization and, most importantly, the in vivo effects of compounds in this class require further investigation.

#### 8.1.4 Compound‐39: Pioneers of the New Frontier

Phenylalanine derivatives and N‐cyano pyrrolidines have enriched the development pipeline for USP30 inhibitors. Among these, the benzosulfonamide‐class, particularly “Compound 39/USP30inh,” stands out due to comprehensive mechanistic studies (Figure 4D: 1). Integrating biochemical and structural analyses, Compound 39's engagement and inhibition of DUBs within SH‐SY5Y cells were validated by activity‐based protein profiling and mass spectrometry. USP30inh's enzyme kinetics suggest a slow‐binding inhibition, potentially indicative of covalent interactions with USP30, leading to significant conformational changes that disrupt ubiquitin attachment, thereby reducing DUB activity [161]. In a recent study, Rusilowicz‐Jones et al. conducted a comprehensive characterization of compound‐39 at both cellular and biological levels. The results indicated that, upon acute mitochondrial depolarization, a 1 μM concentration of compound‐39 restored impaired mitophagy, manifesting a distinctive response to USP30 inhibition characterized by (i) enhanced ubiquitination of TOM20 and SYNJ2BP, (ii) increased accumulation of phosphoubiquitin, and (iii) upregulated basal mitophagy [162]. Notably, compound‐39 significantly increased the turnover of mitochondrial DNA and reinstated mitophagy comparable to control groups in dopaminergic neurons derived from patients with PD carrying mutations in the Parkin gene [162]. Considering that a distinct pool of USP30 exists in peroxisomes apart from mitochondria, the study also explored the effects of compound‐39 in human bone osteosarcoma (U2OS) cells expressing a fluorescent reporter of pexophagy. Compound‐39 enhanced basal pexophagy through USP30 inhibition, providing a novel avenue to enhance peroxisome turnover through pharmacological intervention with a small synthetic molecule [162]. Thus, compound 39 emerges as a promising compound with notable potency and specificity for USP30 compared to other compounds, facilitating mitophagy and pexophagy. It opens up opportunities for further extended investigation into the in vivo effects of these compounds at preclinical levels.

#### 8.1.5 The Horizon of Innovation: The Q14 Peptide and Beyond

The discovery of the Q14 peptide, derived from USP30's transmembrane domain, unveiled a novel mechanism of inhibition, offering a glimpse into future strategies for designing USP30 inhibitors [163]. The Q14 peptide, by engaging in an allosteric autoinhibition mechanism and featuring an LIR motif, demonstrated a dual mechanism for enhancing mitophagy [163]. This breakthrough deepens our understanding of USP30 and signifies a promising avenue for developing precise inhibitors targeting USP30 and other deubiquitinating enzymes. These findings contribute to DUBs drug discovery's evolving landscape and highlight the potential for innovative strategies in neurodegenerative disease management.

As the narrative of USP30 inhibitors unfolds, each development, from Aumdubin to the Q14 peptide, contributes to a tapestry of innovation in the fight against neurodegenerative diseases. These inhibitors stand not merely as molecular entities but as a key to a future where targeted therapies restore cellular harmony, offering new hope for patients afflicted by these devastating conditions. The continued exploration and refinement of USP30 inhibitors promise to unlock new therapeutic potentials, transforming the treatment landscape in the neurodegeneration realm.

### 8.2 Small Molecule Inhibitors of USP13 and USP14

#### 8.2.1 USP13 Inhibitors

Liu and colleagues developed analogues of spautin‐1, which lacks BBB crossing ability, based on 6‐fluoroquinoline, thieno [3,2‐*b*] pyridine, and 3‐nitrocoumarin backbones, including the compound BK50118‐C [128]. These analogues are more potent USP13 inhibitors (IC_50_: 0.42 nM) compared to spautin‐1 and can readily cross the BBB [128]. Interestingly, pharmacological inhibition of USP30 with BK50118‐C promoted the ubiquitination and clearance of α‐Synuclein in mutant α‐synuclein (A53T) transgenic mice [128]. Furthermore, the same group investigated the effects of BK50118‐C in USP13 knockout mice expressing human α‐Synuclein [147]. Their findings demonstrated that BK50118‐C significantly decreased levels of ubiquitinated α‐Synuclein, increased dopamine, and restored behavioral deficits in wild‐type mice, but not in USP13 knockout mice, suggesting the potential of USP13 inhibitor BK50118‐C as a novel therapeutic agent of α‐synucleinopathies [147].

#### 8.2.2 USP14 Inhibitors

Lee and colleagues identified specific small‐molecule inhibitors of human USP14, notably IU1 (1‐[1‐(4‐fluorophenyl)‐2,5‐dimethylpyrrol‐3‐yl]‐2‐pyrrolidin‐1‐ylethanone) through high‐throughput screening [164]. IU1 treatment in mouse embryonic fibroblasts (MEFs) and HEK293 cells promoted degradation of several proteasome substrates associated with neurodegenerative diseases, such as tau, TDP‐43, ATXN3, and glial fibrillary acidic protein (GFAP) [164]. Moreover, IU1 treatment potently decreased the accumulation of oxidized proteins; however, this effect was abolished when a proteasome inhibitor was added, suggesting that IU1 does not prevent the oxidative itself, but rather promotes degradation of proteins that are critical targets of oxidative damage [164]. Another study demonstrated that USP14 knockdown or its pharmacological inhibition by IU1 attenuated mitochondrial dysfunction and locomotor impairments in PINK1 and Parkin mutant Drosophila [134]. Beyond PD, the elevation of USP14 has been implicated in AD as well. IU1‐47 accelerated the degradation of wild‐type tau, the pathological tau mutants P301L and P301S, and the A152T tau variant in primary neuronal cultures [165]. Collectively, above limited but seminal studies suggest that interventions targeting other DUBs such as USP13 and USP14 could serve as a promising therapeutic strategy for neurodegenerative diseases, including PD and AD.

### 8.3 Current and Future Clinical Trials Focusing on USP30 Inhibitors

Building upon the promising results from preclinical studies employing various ubiquitin‐specific protease (USP) inhibitors, particularly those targeting USP30, the field is now transitioning these findings into clinical applications. The successful modulation of USP activity in preclinical models has demonstrated potential therapeutic benefits in neurodegenerative diseases characterized by mitochondrial dysfunction. This progression marks a significant step toward translating molecular insights into viable treatments.

Based on the seminal preclinical findings that demonstrated that MTX325 (previously known as MTX115325) protects dopaminergic neurons by promoting mitophagy, Mission Therapeutics initiated a landmark Phase 1 first‐in‐human clinical trial of MTX325, a selective USP30 inhibitor, early in 2024 (https://missiontherapeutics.com/mission‐therapeutics‐commences‐landmark‐trial‐of‐mtx325‐a‐potential‐disease‐modifying‐treatment‐for‐parkinsons‐disease/). In March 2024, the company has announced the completion of dosing the first cohort of healthy volunteers in multi‐part, adaptive Phase 1 study aimed to evaluate safety, tolerability, pharmacokinetics, and brain penetration of MTX325. The company has now planned for single ascending, multiple dose ascending, and elderly healthy volunteer cohorts throughout 2024. These cohorts aim to comprehensively assess how MTX325 behaves in the body and ensure its safety profile is well‐understood across different populations. To further advance the development of MTX325, the company secured $5.2 M in funding in July 2024, from the Michael J. Fox foundation and Parkinson's UK (https://missiontherapeutics.com/mission‐therapeutics‐awarded‐5‐2m‐from‐the‐michael‐j‐fox‐foundation‐and‐parkinsons‐uk‐to‐advance‐potential‐disease‐modifying‐treatment‐mtx325/). This funding will support a pending Phase 1 clinical trial featuring a 28‐day dosing regimen of the MTX325 in individuals at early stages of PD. The trial aims to assess not only safety and tolerability but also the pharmacokinetic profile and CNS penetration of MTX325 in PD patients. Patient dosing is expected to commence early in 2025.

The initiation of clinical trials for MTX325 highlights the immense therapeutic potential of targeting USP30 in neurodegenerative diseases. By inhibiting USP30, MTX325 enhances mitophagy, facilitating the removal of dysfunctional mitochondria and thereby improving cellular health. This mechanism addresses a root cause of neuronal degeneration rather than merely alleviating symptoms, positioning USP30 inhibitors as potential disease‐modifying agents. Beyond PD, mitochondrial dysfunction is a hallmark of various neurodegenerative disorders, including AD, HD, and Amyotrophic lateral sclerosis (ALS). The success of MTX325 could pave the way for USP30 inhibitors to be investigated in these conditions as well, broadening the impact of this therapeutic strategy.

Moreover, the broader family of USPs offers a rich landscape for therapeutic exploration. Other USPs, such as USP13 and USP14, have also been implicated in neurodegeneration and are potential targets for drug development. Advancements in understanding the specific roles and mechanisms of these enzymes will enable the design of highly selective inhibitors, minimizing off‐target effects and maximizing therapeutic benefits.

## 9 Challenges, Limitations, and Future Directions in the Development of Novel USPs Inhibitors

Despite the promise of USP inhibitors, particularly USP30 inhibitors, several limitations and challenges need to be addressed before they can be widely adopted as therapeutic interventions. While current USP30 inhibitors show specificity for USP30, the possibility of off‐target effects on other DUBs cannot be entirely ruled out. Since DUBs regulate a variety of cellular processes, inhibition at a broader level could perturb critical cellular pathways, leading to side effects. Therefore, future research should focus on improving the selectivity of these inhibitors through advanced structure–activity relationship (SAR) studies and designing inhibitors that specifically target USP30's active site without affecting other closely related DUBs. Furthermore, understanding the precise structural interactions between USP30 and its inhibitors is paramount. Future structural studies using techniques such as X‐ray crystallography or cryo‐electron microscopy are required to provide atomic‐level insights into the binding mechanisms of USP30 inhibitors. These studies could help refine the design of inhibitors to improve specificity, efficacy, and bioavailability, thus optimizing their therapeutic potential.

Ensuring that USP30 inhibitors have acceptable bioavailability, pharmacokinetics, and tissue‐specific targeting represents another challenge. Of note, inhibitors need to efficiently cross the BBB to treat neurodegenerative conditions. Hence, future studies should focus on developing delivery systems that may enhance the penetration of USP30 inhibitors into target tissues and minimize systemic exposure. The inhibition of USP30 might activate compensatory pathways, either through other DUBs or alternative mitophagy regulators, which could reduce the efficacy of the treatment. Thus, it would be worth evaluating whether dual inhibition of USP30 and other compensatory DUBs could yield more pronounced therapeutic benefits, offering new avenues for combination therapies. Future research should also focus on elucidating whether and how these USPs interact or influence each other's activity, which could reveal potential regulatory networks within the ubiquitin‐proteasome system. Despite early preclinical studies indicating the therapeutic potential of USP30 inhibitors, long‐term safety profiles are largely unknown. Chronic downregulation of USP30 could potentially disturb ubiquitin homeostasis or other critical cellular processes, such as mitochondrial dynamics and biogenesis. Comprehensive chronic in vivo studies and early‐stage clinical trials will be required to assess the safety and efficacy of these inhibitors in the context of neurodegenerative diseases. Finally, while preclinical studies have shown promise, translating USP30 inhibitors to human clinical trials will require overcoming several regulatory and experimental hurdles. It will be critical to identify appropriate biomarkers to monitor the efficacy of these inhibitors and determine patient populations most likely to benefit from USP30‐targeted therapies. Furthermore, potential genetic variations in the USP30 gene may influence individual responses to inhibitors, suggesting a need for personalized approaches in future treatments.

## 10 Concluding Remarks

In conclusion, in the dynamic landscape of mitophagy research over the last decade, significant progress has been made in understanding the complex molecular mechanisms that regulate mitochondrial quality control. Among these, deubiquitinating enzymes (DUBs), particularly USP30, have emerged as key players. USP30's distinct affinity for K6‐, K11‐, and K63‐linked ubiquitin chains marks it as a unique contributor to cellular processes like pexophagy, apoptosis, and critically, mitophagy via the PINK1/Parkin‐dependent and PINK1/Parkin‐independent pathway. The implications of USP30 overexpression in neurodegenerative diseases, including AD and PD, signify its potential as a novel target for therapeutic intervention.

Compared to current therapeutic interventions and conventional mitophagy modulators, USP inhibitors offer several advantages in the treatment of neurodegenerative diseases. Traditional therapies primarily focus on symptomatic relief and do not address the underlying mitochondrial dysfunction that contributes to neurodegeneration. Conventional mitophagy modulators often lack specificity, potentially affecting multiple pathways and leading to unintended side effects. USP inhibitors, particularly those targeting USP30, provide a more precise therapeutic approach by directly modulating the deubiquitination processes that impair mitophagy. By inhibiting USP30, these compounds enhance the removal of damaged mitochondria, thereby restoring mitochondrial function and improving cellular health. This mechanism addresses a root cause of neuronal degeneration rather than merely alleviating symptoms, positioning USP inhibitors as potential disease‐modifying agents.

Furthermore, as we encapsulate our exploration into the potential of USP30 inhibitors, it is clear that this class of compounds represents a pivotal advancement in the quest for novel treatments of neurodegenerative diseases. Among them, benzosulfonamide‐class inhibitors, especially “Compound 39/USP30inh,” have demonstrated exceptional specificity and potency against USP30 compared to other DUBs. Their ability to induce mitophagy and pexophagy in both in vitro and in vivo models features their significant therapeutic potential. In addition, MTX325, a novel specific USP30 inhibitor from the N‐cyano pyrrolidine class, also holds promise as it has progressed to clinical trials, marking an important step toward validating its therapeutic efficacy. This compound is currently being evaluated in human studies, further highlighting its potential to restore mitochondrial function in neurodegenerative diseases characterized by mitochondrial dysfunction. This promising avenue offers new hope for restoring mitochondrial function in neurodegenerative diseases marked by mitochondrial dysfunction and opens the door to further preclinical and clinical investigations, positioning USP30 inhibitors as exciting candidates for disease‐modifying treatments.

## Author Contributions

We declare that all authors made fundamental contributions to the manuscript. A.S., H.P., P.P., and A.M.K. conceptualized the review and made table of contents. A.S., H.P., and P.P. prepared the first draft. P.P. prepared the illustrations and compiled the manuscript. P.P. revised the manuscript. P.P. and A.M.K. proofread the manuscript. All authors read and approved the final version of the manuscript.

## Conflicts of Interest

The authors declare no conflicts of interest.

## Data Availability

Data sharing is not applicable to this article as no new data were created or analyzed in this study.

## References

[1] P. Heuveline , “Global and National Declines in Life Expectancy: An End‐Of‐2021 Assessment,” Population and Development Review 48, no. 1 (2022): 31–50. 37325186 10.1111/padr.12477 PMC10270701

[2] D. S. Kehler and O. Theou , “The Impact of Physical Activity and Sedentary Behaviors on Frailty Levels,” Mechanisms of Ageing and Development 180 (2019): 29–41. 30926562 10.1016/j.mad.2019.03.004

[3] D. Melzer , L. C. Pilling , and L. Ferrucci , “The Genetics of Human Ageing,” Nature Reviews Genetics 21, no. 2 (2020): 88–101. 10.1038/s41576-019-0183-6 PMC9934000 31690828

[4] C. López‐Otín and G. Kroemer , “Hallmarks of Health,” Cell 184, no. 1 (2021): 33–63. 33340459 10.1016/j.cell.2020.11.034

[5] D. J. Klionsky , G. Petroni , R. K. Amaravadi , et al., “Autophagy in Major Human Diseases,” EMBO Journal 40, no. 19 (2021): e108863. 34459017 10.15252/embj.2021108863 PMC8488577

[6] T. Vogiatzi , M. Xilouri , K. Vekrellis , and L. Stefanis , “Wild Type α‐Synuclein Is Degraded by Chaperone‐Mediated Autophagy and Macroautophagy in Neuronal Cells,” Journal of Biological Chemistry 283, no. 35 (2008): 23542–23556. 18566453 10.1074/jbc.M801992200 PMC2527094

[7] J. L. Webb , B. Ravikumar , J. Atkins , J. N. Skepper , and D. C. Rubinsztein , “α‐Synuclein Is Degraded by Both Autophagy and the Proteasome,” Journal of Biological Chemistry 278, no. 27 (2003): 25009–25013. 12719433 10.1074/jbc.M300227200

[8] M. Xilouri , T. Vogiatzi , and L. Stefanis , “Alpha‐Synuclein Degradation by Autophagic Pathways: A Potential Key to Parkinson's Disease Pathogenesis,” Autophagy 4, no. 7 (2008): 917–919. 18708765 10.4161/auto.6685

[9] Y. Wang , U. Krüger , E. Mandelkow , and E.‐M. Mandelkow , “Generation of Tau Aggregates and Clearance by Autophagy in an Inducible Cell Model of Tauopathy,” Neurodegenerative Diseases 7, no. 1–3 (2010): 103–107. 20173337 10.1159/000285516

[10] B. Ravikumar , R. Duden , and D. C. Rubinsztein , “Aggregate‐Prone Proteins With Polyglutamine and Polyalanine Expansions Are Degraded by Autophagy,” Human Molecular Genetics 11, no. 9 (2002): 1107–1117. 11978769 10.1093/hmg/11.9.1107

[11] S. Sarkar , J. E. Davies , Z. Huang , A. Tunnacliffe , and D. C. Rubinsztein , “Trehalose, a Novel mTOR‐Independent Autophagy Enhancer, Accelerates the Clearance of Mutant Huntingtin and α‐Synuclein,” Journal of Biological Chemistry 282, no. 8 (2007): 5641–5652. 17182613 10.1074/jbc.M609532200

[12] M. Shibata , T. Lu , T. Furuya , et al., “Regulation of Intracellular Accumulation of Mutant Huntingtin by Beclin 1,” Journal of Biological Chemistry 281, no. 20 (2006): 14474–14485. 16522639 10.1074/jbc.M600364200

[13] P. Parekh , N. Sharma , A. Gadepalli , A. Shahane , M. Sharma , and A. Khairnar , “A Cleaning Crew: The Pursuit of Autophagy in Parkinson's Disease,” ACS Chemical Neuroscience 10, no. 9 (2019): 3914–3926. 31385687 10.1021/acschemneuro.9b00244

[14] F. M. Menzies , A. Fleming , A. Caricasole , et al., “Autophagy and Neurodegeneration: Pathogenic Mechanisms and Therapeutic Opportunities,” Neuron 93, no. 5 (2017): 1015–1034. 28279350 10.1016/j.neuron.2017.01.022

[15] Y. Ohsumi , “Historical Landmarks of Autophagy Research,” Cell Research 24, no. 1 (2014): 9–23. 24366340 10.1038/cr.2013.169 PMC3879711

[16] W. Li , P. He , Y. Huang , et al., “Selective Autophagy of Intracellular Organelles: Recent Research Advances,” Theranostics 11, no. 1 (2021): 222–256. 33391472 10.7150/thno.49860 PMC7681076

[17] Y. Chen , Z. Zhou , and W. Min , “Mitochondria, Oxidative Stress and Innate Immunity,” Frontiers in Physiology 9 (2018): 1487. 30405440 10.3389/fphys.2018.01487 PMC6200916

[18] S. Dadsena , C. Zollo , and A. J. García‐Sáez , “Mechanisms of Mitochondrial Cell Death,” Biochemical Society Transactions 49, no. 2 (2021): 663–674. 33704419 10.1042/BST20200522

[19] M. Lewis and W. Lewis , “Mitochondria in Tissue Culture,” Science 39, no. 1000 (1914): 330–333. 17794648 10.1126/science.39.1000.330

[20] R. Kesharwani , D. Sarmah , H. Kaur , et al., “Interplay Between Mitophagy and Inflammasomes in Neurological Disorders,” ACS Chemical Neuroscience 10, no. 5 (2019): 2195–2208. 30917655 10.1021/acschemneuro.9b00117

[21] R. K. Dagda , S. J. Cherra , S. M. Kulich , A. Tandon , D. Park , and C. T. Chu , “Loss of PINK1 Function Promotes Mitophagy Through Effects on Oxidative Stress and Mitochondrial Fission,” Journal of Biological Chemistry 284, no. 20 (2009): 13843–13855. 19279012 10.1074/jbc.M808515200 PMC2679485

[22] D. Narendra , A. Tanaka , D.‐F. Suen , and R. J. Youle , “Parkin Is Recruited Selectively to Impaired Mitochondria and Promotes Their Autophagy,” Journal of Cell Biology 183, no. 5 (2008): 795–803. 19029340 10.1083/jcb.200809125 PMC2592826

[23] A. B. Malpartida , M. Williamson , D. P. Narendra , R. Wade‐Martins , and B. J. Ryan , “Mitochondrial Dysfunction and Mitophagy in Parkinson's Disease: From Mechanism to Therapy,” Trends in Biochemical Sciences 46, no. 4 (2021): 329–343. 33323315 10.1016/j.tibs.2020.11.007

[24] J. A. Pradeepkiran and P. H. Reddy , “Defective Mitophagy in Alzheimer's Disease,” Ageing Research Reviews 64 (2020): 101191. 33022416 10.1016/j.arr.2020.101191 PMC7710581

[25] M. Funayama , K. Nishioka , Y. Li , and N. Hattori , “Molecular Genetics of Parkinson's Disease: Contributions and Global Trends,” Journal of Human Genetics 68, no. 3 (2023): 125–130. 35821405 10.1038/s10038-022-01058-5 PMC9968657

[26] T. Kitada , Y. Tong , C. A. Gautier , and J. Shen , “Absence of Nigral Degeneration in Aged Parkin/DJ‐1/PINK1 Triple Knockout Mice,” Journal of Neurochemistry 111, no. 3 (2009): 696–702. 19694908 10.1111/j.1471-4159.2009.06350.x PMC2952933

[27] F. B. Gonçalves and V. A. Morais , “PINK1: A Bridge Between Mitochondria and Parkinson's Disease,” Life 11, no. 5 (2021): 371. 33919398 10.3390/life11050371 PMC8143285

[28] S. M. Jin and R. J. Youle , “PINK1‐and Parkin‐Mediated Mitophagy at a Glance,” Journal of Cell Science 125, no. 4 (2012): 795–799. 22448035 10.1242/jcs.093849 PMC3656616

[29] S. M. Jin , M. Lazarou , C. Wang , L. A. Kane , D. P. Narendra , and R. J. Youle , “Mitochondrial Membrane Potential Regulates PINK1 Import and Proteolytic Destabilization by PARL,” Journal of Cell Biology 191, no. 5 (2010): 933–942. 21115803 10.1083/jcb.201008084 PMC2995166

[30] A. Varshavsky , “N‐Degron and C‐Degron Pathways of Protein Degradation,” Proceedings of the National Academy of Sciences of the United States of America 116, no. 2 (2019): 358–366. 30622213 10.1073/pnas.1816596116 PMC6329975

[31] R. J. Youle and D. P. Narendra , “Mechanisms of Mitophagy,” Nature Reviews Molecular Cell Biology 12, no. 1 (2011): 9–14. 21179058 10.1038/nrm3028 PMC4780047

[32] S. Akabane , K. Watanabe , H. Kosako , et al., “TIM23 Facilitates PINK1 Activation by Safeguarding Against OMA1‐Mediated Degradation in Damaged Mitochondria,” Cell Reports 42, no. 5 (2023): 112454. 37160114 10.1016/j.celrep.2023.112454

[33] M. A. Eldeeb , A. N. Bayne , A. Fallahi , et al., “Tom20 Gates PINK1 Activity and Mediates Its Tethering of the TOM and TIM23 Translocases Upon Mitochondrial Stress,” Proceedings of the National Academy of Sciences of the United States of America 121, no. 10 (2024): e2313540121. 38416681 10.1073/pnas.2313540121 PMC10927582

[34] V. Sauvé , G. Sung , N. Soya , et al., “Mechanism of Parkin Activation by Phosphorylation,” Nature Structural & Molecular Biology 25, no. 7 (2018): 623–630. 10.1038/s41594-018-0088-7 29967542

[35] A. Kazlauskaite , R. J. Martínez‐Torres , S. Wilkie , et al., “Binding to Serine 65‐Phosphorylated Ubiquitin Primes Parkin for Optimal PINK 1‐Dependent Phosphorylation and Activation,” EMBO Reports 16, no. 8 (2015): 939–954. 26116755 10.15252/embr.201540352 PMC4552487

[36] A. Kumar , J. D. Aguirre , T. E. Condos , et al., “Disruption of the Autoinhibited State Primes the E3 Ligase Parkin for Activation and Catalysis,” EMBO Journal 34, no. 20 (2015): 2506–2521. 26254304 10.15252/embj.201592337 PMC4609183

[37] V. Sauvé , A. Lilov , M. Seirafi , et al., “A Ubl/Ubiquitin Switch in the Activation of Parkin,” EMBO Journal 34, no. 20 (2015): 2492–2505. 26254305 10.15252/embj.201592237 PMC4609182

[38] T. Wauer , M. Simicek , A. Schubert , and D. Komander , “Mechanism of Phospho‐Ubiquitin‐Induced PARKIN Activation,” Nature 524, no. 7565 (2015): 370–374. 26161729 10.1038/nature14879 PMC4984986

[39] K. Yamano , B. B. Queliconi , F. Koyano , et al., “Site‐Specific Interaction Mapping of Phosphorylated Ubiquitin to Uncover Parkin Activation,” Journal of Biological Chemistry 290, no. 42 (2015): 25199–25211. 26260794 10.1074/jbc.M115.671446 PMC4646171

[40] T. Wauer , K. N. Swatek , J. L. Wagstaff , et al., “Ubiquitin Ser65 Phosphorylation Affects Ubiquitin Structure, Chain Assembly and Hydrolysis,” EMBO Journal 34, no. 3 (2015): 307–325. 25527291 10.15252/embj.201489847 PMC4339119

[41] V. Kirkin , T. Lamark , Y.‐S. Sou , et al., “A Role for NBR1 in Autophagosomal Degradation of Ubiquitinated Substrates,” Molecular Cell 33, no. 4 (2009): 505–516. 19250911 10.1016/j.molcel.2009.01.020

[42] M. Lazarou , D. A. Sliter , L. A. Kane , et al., “The Ubiquitin Kinase PINK1 Recruits Autophagy Receptors to Induce Mitophagy,” Nature 524, no. 7565 (2015): 309–314. 26266977 10.1038/nature14893 PMC5018156

[43] T. L. Thurston , G. Ryzhakov , S. Bloor , N. Von Muhlinen , and F. Randow , “The TBK1 Adaptor and Autophagy Receptor NDP52 Restricts the Proliferation of Ubiquitin‐Coated Bacteria,” Nature Immunology 10, no. 11 (2009): 1215–1221. 19820708 10.1038/ni.1800

[44] Y. C. Wong and E. L. Holzbaur , “Optineurin Is an Autophagy Receptor for Damaged Mitochondria in Parkin‐Mediated Mitophagy That Is Disrupted by an ALS‐Linked Mutation,” Proceedings of the National Academy of Sciences of the United States of America 111, no. 42 (2014): E4439–E4448. 25294927 10.1073/pnas.1405752111 PMC4210283

[45] S. Geisler , K. M. Holmström , D. Skujat , et al., “PINK1/Parkin‐Mediated Mitophagy Is Dependent on VDAC1 and p62/SQSTM1,” Nature Cell Biology 12, no. 2 (2010): 119–131. 20098416 10.1038/ncb2012

[46] S. A. Sarraf , M. Raman , V. Guarani‐Pereira , et al., “Landscape of the PARKIN‐Dependent Ubiquitylome in Response to Mitochondrial Depolarization,” Nature 496, no. 7445 (2013): 372–376. 23503661 10.1038/nature12043 PMC3641819

[47] D. G. McEwan , D. Popovic , A. Gubas , et al., “PLEKHM1 Regulates Autophagosome‐Lysosome Fusion Through HOPS Complex and LC3/GABARAP Proteins,” Molecular Cell 57, no. 1 (2015): 39–54. 25498145 10.1016/j.molcel.2014.11.006

[48] C. N. Cunningham , J. M. Baughman , L. Phu , et al., “USP30 and Parkin Homeostatically Regulate Atypical Ubiquitin Chains on Mitochondria,” Nature Cell Biology 17, no. 2 (2015): 160–169. 25621951 10.1038/ncb3097

[49] R. Iorio , G. Celenza , and S. Petricca , “Mitophagy: Molecular Mechanisms, New Concepts on Parkin Activation and the Emerging Role of AMPK/ULK1 Axis,” Cells 11, no. 1 (2021): 30. 35011593 10.3390/cells11010030 PMC8750607

[50] T. G. McWilliams , A. R. Prescott , L. Montava‐Garriga , et al., “Basal Mitophagy Occurs Independently of PINK1 in Mouse Tissues of High Metabolic Demand,” Cell Metabolism 27, no. 2 (2018): 439–449.e435. 29337137 10.1016/j.cmet.2017.12.008 PMC5807059

[51] J. J. Lee , A. Sanchez‐Martinez , A. M. Zarate , et al., “Basal Mitophagy Is Widespread in Drosophila but Minimally Affected by Loss of Pink1 or Parkin,” Journal of Cell Biology 217, no. 5 (2018): 1613–1622. 29500189 10.1083/jcb.201801044 PMC5940313

[52] J. Liu , W. Liu , R. Li , and H. Yang , “Mitophagy in Parkinson's Disease: From Pathogenesis to Treatment,” Cells 8, no. 7 (2019): 712. 31336937 10.3390/cells8070712 PMC6678174

[53] A. Diwan , M. Krenz , F. M. Syed , et al., “Inhibition of Ischemic Cardiomyocyte Apoptosis Through Targeted Ablation of Bnip3 Restrains Postinfarction Remodeling in Mice,” Journal of Clinical Investigation 117, no. 10 (2007): 2825–2833. 17909626 10.1172/JCI32490 PMC1994631

[54] A. Diwan , S. J. Matkovich , Q. Yuan , et al., “Endoplasmic Reticulum–Mitochondria Crosstalk in NIX‐Mediated Murine Cell Death,” Journal of Clinical Investigation 119, no. 1 (2009): 203–212. 19065046 10.1172/JCI36445 PMC2613462

[55] N. Ohi , A. Tokunaga , H. Tsunoda , et al., “A Novel Adenovirus E1B19K‐Binding Protein B5 Inhibits Apoptosis Induced by Nip3 by Forming a Heterodimer Through the C‐Terminal Hydrophobic Region,” Cell Death and Differentiation 6, no. 4 (1999): 314–325. 10381623 10.1038/sj.cdd.4400493

[56] M. Yasuda , J.‐w. Han , C. A. Dionne , J. M. Boyd , and G. Chinnadurai , “BNIP3α: A Human Homolog of Mitochondrial Proapoptotic Protein BNIP3,” Cancer Research 59, no. 3 (1999): 533–537. 9973195

[57] R. A. Hanna , M. N. Quinsay , A. M. Orogo , K. Giang , S. Rikka , and Å. B. Gustafsson , “Microtubule‐Associated Protein 1 Light Chain 3 (LC3) Interacts With Bnip3 Protein to Selectively Remove Endoplasmic Reticulum and Mitochondria via Autophagy,” Journal of Biological Chemistry 287, no. 23 (2012): 19094–19104. 22505714 10.1074/jbc.M111.322933 PMC3365942

[58] W. Mughal , L. Nguyen , S. Pustylnik , et al., “A Conserved MADS‐Box Phosphorylation Motif Regulates Differentiation and Mitochondrial Function in Skeletal, Cardiac, and Smooth Muscle Cells,” Cell Death & Disease 6, no. 10 (2015): e1944. 26512955 10.1038/cddis.2015.306 PMC5399178

[59] S. C. da Silva Rosa , M. D. Martens , J. T. Field , et al., “BNIP3L/nix‐Induced Mitochondrial Fission, Mitophagy, and Impaired Myocyte Glucose Uptake Are Abrogated by PRKA/PKA Phosphorylation,” Autophagy 17, no. 9 (2021): 2257–2272. 33044904 10.1080/15548627.2020.1821548 PMC8496715

[60] Y. Ren , J. Chen , X. Wu , et al., “Role of c‐Abl‐GSK3β Signaling in MPP+‐Induced Autophagy‐Lysosomal Dysfunction,” Toxicological Sciences 165, no. 1 (2018): 232–243. 30165626 10.1093/toxsci/kfy155

[61] H. Li , A. Ham , T. C. Ma , et al., “Mitochondrial Dysfunction and Mitophagy Defect Triggered by Heterozygous GBA Mutations,” Autophagy 15, no. 1 (2019): 113–130. 30160596 10.1080/15548627.2018.1509818 PMC6287702

[62] L. Liu , D. Feng , G. Chen , et al., “Mitochondrial Outer‐Membrane Protein FUNDC1 Mediates Hypoxia‐Induced Mitophagy in Mammalian Cells,” Nature Cell Biology 14, no. 2 (2012): 177–185. 22267086 10.1038/ncb2422

[63] M. Lv , C. Wang , F. Li , et al., “Structural Insights Into the Recognition of Phosphorylated FUNDC1 by LC3B in Mitophagy,” Protein & Cell 8, no. 1 (2017): 25–38. 27757847 10.1007/s13238-016-0328-8 PMC5233613

[64] H. Wu , D. Xue , G. Chen , et al., “The BCL2L1 and PGAM5 Axis Defines Hypoxia‐Induced Receptor‐Mediated Mitophagy,” Autophagy 10, no. 10 (2014): 1712–1725. 25126723 10.4161/auto.29568 PMC4198357

[65] M. Chen , Z. Chen , Y. Wang , et al., “Mitophagy Receptor FUNDC1 Regulates Mitochondrial Dynamics and Mitophagy,” Autophagy 12, no. 4 (2016): 689–702. 27050458 10.1080/15548627.2016.1151580 PMC4836026

[66] A. Di Rita , A. Peschiaroli , P. D′ Acunzo , et al., “HUWE1 E3 Ligase Promotes PINK1/PARKIN‐Independent Mitophagy by Regulating AMBRA1 Activation via IKKα,” Nature Communications 9, no. 1 (2018): 3755. 10.1038/s41467-018-05722-3 PMC6138665 30217973

[67] F. Strappazzon , F. Nazio , M. Corrado , et al., “AMBRA1 Is Able to Induce Mitophagy via LC3 Binding, Regardless of PARKIN and p62/SQSTM1,” Cell Death and Differentiation 22, no. 3 (2015): 419–432. 25215947 10.1038/cdd.2014.139 PMC4326570

[68] J. M. Fuentes and P. Morcillo , “The Role of Cardiolipin in Mitochondrial Function and Neurodegenerative Diseases,” Cells 13, no. 7 (2024): 609. 38607048 10.3390/cells13070609 PMC11012098

[69] X.‐X. Li , B. Tsoi , Y.‐F. Li , H. Kurihara , and R.‐R. He , “Cardiolipin and Its Different Properties in Mitophagy and Apoptosis,” Journal of Histochemistry and Cytochemistry 63, no. 5 (2015): 301–311. 25673287 10.1369/0022155415574818 PMC4409943

[70] C. T. Chu , J. Ji , R. K. Dagda , et al., “Cardiolipin Externalization to the Outer Mitochondrial Membrane Acts as an Elimination Signal for Mitophagy in Neuronal Cells,” Nature Cell Biology 15, no. 10 (2013): 1197–1205. 24036476 10.1038/ncb2837 PMC3806088

[71] M. El‐Hafidi , F. Correa , and C. Zazueta , “Mitochondrial Dysfunction in Metabolic and Cardiovascular Diseases Associated With Cardiolipin Remodeling,” Biochimica et Biophysica Acta (BBA) ‐ Molecular Basis of Disease 1866, no. 6 (2020): 165744. 32105822 10.1016/j.bbadis.2020.165744

[72] T. Ryan , V. V. Bamm , M. G. Stykel , et al., “Cardiolipin Exposure on the Outer Mitochondrial Membrane Modulates α‐Synuclein,” Nature Communications 9, no. 1 (2018): 817. 10.1038/s41467-018-03241-9 PMC5827019 29483518

[73] N. Sun , R. J. Youle , and T. Finkel , “The Mitochondrial Basis of Aging,” Molecular Cell 61, no. 5 (2016): 654–666. 26942670 10.1016/j.molcel.2016.01.028 PMC4779179

[74] R. M. Koffie , B. T. Hyman , and T. L. Spires‐Jones , “Alzheimer's Disease: Synapses Gone Cold,” Molecular Neurodegeneration 6, no. 1 (2011): 1–9. 21871088 10.1186/1750-1326-6-63 PMC3178498

[75] M. Zvěřová , “Clinical Aspects of Alzheimer's Disease,” Clinical Biochemistry 72 (2019): 3–6. 31034802 10.1016/j.clinbiochem.2019.04.015

[76] R. M. Ana , B. D. José , R. Fernando , and S. Renata , “Alzheimer's Disease: Insights and New Prospects in Disease Pathophysiology, Biomarkers and Disease‐Modifying Drugs,” Biochemical Pharmacology 115522 (2023): 115522. 10.1016/j.bcp.2023.115522 36996971

[77] M. Manczak and P. H. Reddy , “Abnormal Interaction of VDAC1 With Amyloid Beta and Phosphorylated Tau Causes Mitochondrial Dysfunction in Alzheimer's Disease,” Human Molecular Genetics 21, no. 23 (2012): 5131–5146. 22926141 10.1093/hmg/dds360 PMC3490521

[78] H. Xie , J. Guan , L. A. Borrelli , J. Xu , A. Serrano‐Pozo , and B. J. Bacskai , “Mitochondrial Alterations Near Amyloid Plaques in an Alzheimer's Disease Mouse Model,” Journal of Neuroscience 33, no. 43 (2013): 17042–17051. 24155308 10.1523/JNEUROSCI.1836-13.2013 PMC3807029

[79] H. Du , L. Guo , S. Yan , A. A. Sosunov , G. M. McKhann , and S. ShiDu Yan , “Early Deficits in Synaptic Mitochondria in an Alzheimer's Disease Mouse Model,” Proceedings of the National Academy of Sciences of the United States of America 107, no. 43 (2010): 18670–18675. 20937894 10.1073/pnas.1006586107 PMC2972922

[80] J. L. Bayer‐Carter , P. S. Green , T. J. Montine , et al., “Diet Intervention and Cerebrospinal Fluid Biomarkers in Amnestic Mild Cognitive Impairment,” Archives of Neurology 68, no. 6 (2011): 743–752. 21670398 10.1001/archneurol.2011.125 PMC3175115

[81] R. B. Speisman , A. Kumar , A. Rani , T. C. Foster , and B. K. Ormerod , “Daily Exercise Improves Memory, Stimulates Hippocampal Neurogenesis and Modulates Immune and Neuroimmune Cytokines in Aging Rats,” Brain, Behavior, and Immunity 28 (2013): 25–43. 23078985 10.1016/j.bbi.2012.09.013 PMC3545095

[82] S. S. Karuppagounder , S. Brahmachari , Y. Lee , V. L. Dawson , T. M. Dawson , and H. S. Ko , “The c‐Abl Inhibitor, Nilotinib, Protects Dopaminergic Neurons in a Preclinical Animal Model of Parkinson's Disease,” Scientific Reports 4, no. 1 (2014): 4874. 24786396 10.1038/srep04874 PMC4007078

[83] J. Qiu , Y. Chen , J. Zhuo , et al., “Urolithin A Promotes Mitophagy and Suppresses NLRP3 Inflammasome Activation in Lipopolysaccharide‐Induced BV2 Microglial Cells and MPTP‐Induced Parkinson's Disease Model,” Neuropharmacology 207 (2022): 108963. 35065082 10.1016/j.neuropharm.2022.108963

[84] E. F. Fang , Y. Hou , K. Palikaras , et al., “Mitophagy Inhibits Amyloid‐β and Tau Pathology and Reverses Cognitive Deficits in Models of Alzheimer's Disease,” Nature Neuroscience 22, no. 3 (2019): 401–412. 30742114 10.1038/s41593-018-0332-9 PMC6693625

[85] W. Poewe , K. Seppi , C. M. Tanner , et al., “Parkinson Disease,” Nature Reviews Disease Primers 3, no. 1 (2017): 1–21. 10.1038/nrdp.2017.13 28332488

[86] B. R. Bloem , M. S. Okun , and C. Klein , “Parkinson's Disease,” Lancet 397, no. 10291 (2021): 2284–2303. 33848468 10.1016/S0140-6736(21)00218-X

[87] W.‐S. Choi , R. D. Palmiter , and Z. Xia , “Loss of Mitochondrial Complex I Activity Potentiates Dopamine Neuron Death Induced by Microtubule Dysfunction in a Parkinson's Disease Model,” Journal of Cell Biology 192, no. 5 (2011): 873–882. 21383081 10.1083/jcb.201009132 PMC3051820

[88] D. Ramonet , C. Perier , A. Recasens , et al., “Optic Atrophy 1 Mediates Mitochondria Remodeling and Dopaminergic Neurodegeneration Linked to Complex I Deficiency,” Cell Death and Differentiation 20, no. 1 (2013): 77–85. 22858546 10.1038/cdd.2012.95 PMC3524632

[89] B. Wang , N. Abraham , G. Gao , and Q. Yang , “Dysregulation of Autophagy and Mitochondrial Function in Parkinson's Disease,” Translational Neurodegeneration 5, no. 1 (2016): 1–9. 27822367 10.1186/s40035-016-0065-1 PMC5087125

[90] R. M. Ivatt , A. Sanchez‐Martinez , V. K. Godena , S. Brown , E. Ziviani , and A. J. Whitworth , “Genome‐Wide RNAi Screen Identifies the Parkinson Disease GWAS Risk Locus SREBF1 as a Regulator of Mitophagy,” Proceedings of the National Academy of Sciences of the United States of America 111, no. 23 (2014): 8494–8499. 24912190 10.1073/pnas.1321207111 PMC4060696

[91] R. M. Ivatt and A. J. Whitworth , “SREBF1 Links Lipogenesis to Mitophagy and Sporadic Parkinson Disease,” Autophagy 10, no. 8 (2014): 1476–1477. 24991824 10.4161/auto.29642 PMC4203527

[92] V. Bonifati , “Autosomal Recessive Parkinsonism,” Parkinsonism & Related Disorders 18 (2012): S4–S6. 22166450 10.1016/S1353-8020(11)70004-9

[93] K. J. Thomas , M. K. McCoy , J. Blackinton , et al., “DJ‐1 Acts in Parallel to the PINK1/Parkin Pathway to Control Mitochondrial Function and Autophagy,” Human Molecular Genetics 20, no. 1 (2011): 40–50. 20940149 10.1093/hmg/ddq430 PMC3000675

[94] S. Ishikawa , T. Taira , T. Niki , et al., “Oxidative Status of DJ‐1‐Dependent Activation of Dopamine Synthesis Through Interaction of Tyrosine Hydroxylase and 4‐Dihydroxy‐L‐Phenylalanine (L‐DOPA) Decarboxylase With DJ‐1,” Journal of Biological Chemistry 284, no. 42 (2009): 28832–28844. 19703902 10.1074/jbc.M109.019950 PMC2781429

[95] H. Ariga , K. Takahashi‐Niki , I. Kato , H. Maita , T. Niki , and S. M. Iguchi‐Ariga , “Neuroprotective Function of DJ‐1 in Parkinson's Disease,” Oxidative Medicine and Cellular Longevity 2013 (2013): 683920. 23766857 10.1155/2013/683920 PMC3671546

[96] K. Takahashi‐Niki , T. Niki , S. M. Iguchi‐Ariga , and H. Ariga , “Transcriptional Regulation of DJ‐1,” in DJ‐1/PARK7 Protein: Parkinson's Disease, Cancer and Oxidative Stress‐Induced Diseases (Springer Nature, 2017), 89–95. 10.1007/978-981-10-6583-5_7 29147905

[97] M. K. McCoy and M. R. Cookson , “DJ‐1 Regulation of Mitochondrial Function and Autophagy Through Oxidative Stress,” Autophagy 7, no. 5 (2011): 531–532. 21317550 10.4161/auto.7.5.14684 PMC3127213

[98] N. P. Marotta , J. Ara , N. Uemura , et al., “Alpha‐Synuclein From Patient Lewy Bodies Exhibits Distinct Pathological Activity That Can Be Propagated In Vitro,” Acta Neuropathologica Communications 9, no. 1 (2021): 1–18. 34819159 10.1186/s40478-021-01288-2 PMC8611971

[99] X. Wang , K. Becker , N. Levine , et al., “Pathogenic Alpha‐Synuclein Aggregates Preferentially Bind to Mitochondria and Affect Cellular Respiration,” Acta Neuropathologica Communications 7 (2019): 1–14. 10.1186/s40478-019-0696-4 PMC6419482 30871620

[100] S. Ghio , A. Camilleri , M. Caruana , et al., “Cardiolipin Promotes Pore‐Forming Activity of Alpha‐Synuclein Oligomers in Mitochondrial Membranes,” ACS Chemical Neuroscience 10, no. 8 (2019): 3815–3829. 31356747 10.1021/acschemneuro.9b00320

[101] H. Deng , H. Liang , and J. Jankovic , “F‐Box Only Protein 7 Gene in Parkinsonian‐Pyramidal Disease,” JAMA Neurology 70, no. 1 (2013): 20–24. 23318512 10.1001/jamaneurol.2013.572

[102] E. Lohmann , A. S. Coquel , A. Honoré , et al., “A New F‐Box Protein 7 Gene Mutation Causing Typical Parkinson's Disease,” Movement Disorders 30, no. 8 (2015): 1130–1133. 26010069 10.1002/mds.26266

[103] D. E. Nelson , S. J. Randle , and H. Laman , “Beyond Ubiquitination: The Atypical Functions of Fbxo7 and Other F‐Box Proteins,” Open Biology 3, no. 10 (2013): 130131. 24107298 10.1098/rsob.130131 PMC3814724

[104] F. Rey , S. Ottolenghi , G. V. Zuccotti , M. Samaja , and S. Carelli , “Mitochondrial Dysfunctions in Neurodegenerative Diseases: Role in Disease Pathogenesis, Strategies for Analysis and Therapeutic Prospects,” Neural Regeneration Research 17, no. 4 (2022): 754–758. 34472461 10.4103/1673-5374.322430 PMC8530118

[105] S. Kshirsagar , N. Sawant , H. Morton , A. P. Reddy , and P. H. Reddy , “Mitophagy Enhancers Against Phosphorylated Tau‐Induced Mitochondrial and Synaptic Toxicities in Alzheimer Disease,” Pharmacological Research 174 (2021): 105973. 34763094 10.1016/j.phrs.2021.105973 PMC8670983

[106] N. Varghese , S. Werner , A. Grimm , and A. Eckert , “Dietary Mitophagy Enhancer: A Strategy for Healthy Brain Aging?,” Antioxidants 9, no. 10 (2020): 932. 33003315 10.3390/antiox9100932 PMC7600282

[107] I. Lonskaya , M. L. Hebron , N. M. Desforges , J. B. Schachter , and C. E. Moussa , “Nilotinib‐Induced Autophagic Changes Increase Endogenous Parkin Level and Ubiquitination, Leading to Amyloid Clearance,” Journal of Molecular Medicine 92 (2014): 373–386. 24337465 10.1007/s00109-013-1112-3 PMC3975659

[108] C.‐H. Hsieh , L. Li , R. Vanhauwaert , et al., “Miro1 Marks Parkinson's Disease Subset and Miro1 Reducer Rescues Neuron Loss in Parkinson's Models,” Cell Metabolism 30, no. 6 (2019): 1131–1140.e7. 31564441 10.1016/j.cmet.2019.08.023 PMC6893131

[109] H. Wang , J. Fu , X. Xu , Z. Yang , and T. Zhang , “Rapamycin Activates Mitophagy and Alleviates Cognitive and Synaptic Plasticity Deficits in a Mouse Model of Alzheimer's Disease,” Journals of Gerontology: Series A 76, no. 10 (2021): 1707–1713. 10.1093/gerona/glab142 34003967

[110] I. Maestro , L. R. De la Ballina , A. Simonsen , P. Boya , and A. Martinez , “Phenotypic Assay Leads to Discovery of Mitophagy Inducers With Therapeutic Potential for Parkinson's Disease,” ACS Chemical Neuroscience 12, no. 24 (2021): 4512–4523. 34846852 10.1021/acschemneuro.1c00529

[111] K. Shiba‐Fukushima , T. Inoshita , O. Sano , et al., “A Cell‐Based High‐Throughput Screening Identified Two Compounds That Enhance PINK1‐Parkin Signaling,” Iscience 23 (2020): 23 (5). 10.1016/j.isci.2020.101048 PMC7183160 32335362

[112] D.‐X. Wang , Y. Yang , X.‐S. Huang , et al., “Pramipexole Attenuates Neuronal Injury in Parkinson's Disease by Targeting miR‐96 to Activate BNIP3‐Mediated Mitophagy,” Neurochemistry International 146 (2021): 104972. 33493581 10.1016/j.neuint.2021.104972

[113] N. Moskal , V. Riccio , M. Bashkurov , et al., “ROCK Inhibitors Upregulate the Neuroprotective Parkin‐Mediated Mitophagy Pathway,” Nature Communications 11, no. 1 (2020): 88. 10.1038/s41467-019-13781-3 PMC6941965 31900402

[114] Y. Liu , T. B. Lear , M. Verma , et al., “Chemical Inhibition of FBXO7 Reduces Inflammation and Confers Neuroprotection by Stabilizing the Mitochondrial Kinase PINK1,” JCI Insight 5, no. 11 (2020): e131834. 32493843 10.1172/jci.insight.131834 PMC7308049

[115] F. Singh , A. R. Prescott , P. Rosewell , G. Ball , A. D. Reith , and I. G. Ganley , “Pharmacological Rescue of Impaired Mitophagy in Parkinson's Disease‐Related LRRK2 G2019S Knock‐In Mice,” eLife 10 (2021): e67604. 34340748 10.7554/eLife.67604 PMC8331189

[116] H. S. Ko , Y. Lee , J.‐H. Shin , et al., “Phosphorylation by the c‐Abl Protein Tyrosine Kinase Inhibits Parkin's Ubiquitination and Protective Function,” Proceedings of the National Academy of Sciences of the United States of America 107, no. 38 (2010): 16691–16696. 20823226 10.1073/pnas.1006083107 PMC2944759

[117] C.‐H. Hsieh , A. Shaltouki , A. E. Gonzalez , et al., “Functional Impairment in Miro Degradation and Mitophagy Is a Shared Feature in Familial and Sporadic Parkinson's Disease,” Cell Stem Cell 19, no. 6 (2016): 709–724. 27618216 10.1016/j.stem.2016.08.002 PMC5135570

[118] J. B. Mannick and D. W. Lamming , “Targeting the Biology of Aging With mTOR Inhibitors,” Nature Aging 3 (2023): 1–19. 37142830 10.1038/s43587-023-00416-y PMC10330278

[119] B. Bingol and M. Sheng , “Mechanisms of Mitophagy: PINK1, Parkin, USP30 and Beyond,” Free Radical Biology and Medicine 100 (2016): 210–222. 27094585 10.1016/j.freeradbiomed.2016.04.015

[120] B. Bingol , J. S. Tea , L. Phu , et al., “The Mitochondrial Deubiquitinase USP30 Opposes Parkin‐Mediated Mitophagy,” Nature 510, no. 7505 (2014): 370–375. 24896179 10.1038/nature13418

[121] T. Cornelissen , D. Haddad , F. Wauters , et al., “The Deubiquitinase USP15 Antagonizes Parkin‐Mediated Mitochondrial Ubiquitination and Mitophagy,” Human Molecular Genetics 23, no. 19 (2014): 5227–5242. 24852371 10.1093/hmg/ddu244 PMC7108632

[122] T. M. Durcan and E. A. Fon , “The Three ‘P's of Mitophagy: PARKIN, PINK1, and Post‐Translational Modifications,” Genes & Development 29, no. 10 (2015): 989–999. 25995186 10.1101/gad.262758.115 PMC4441056

[123] Y. Wang , M. Serricchio , M. Jauregui , et al., “Deubiquitinating Enzymes Regulate PARK2‐Mediated Mitophagy,” Autophagy 11, no. 4 (2015): 595–606. 25915564 10.1080/15548627.2015.1034408 PMC4502823

[124] H. Peng , F. Yang , Q. Hu , et al., “The Ubiquitin‐Specific Protease USP8 Directly Deubiquitinates SQSTM1/p62 to Suppress Its Autophagic Activity,” Autophagy 16, no. 4 (2020): 698–708. 31241013 10.1080/15548627.2019.1635381 PMC7138243

[125] J. Santelices , M. Ou , G. H. Maegawa , K. Hercik , and M. J. Edelmann , “USP8 Inhibition Regulates Autophagy Flux and Controls Salmonella Infection,” Frontiers in Cellular and Infection Microbiology 13 (2023): 1070271. 37026055 10.3389/fcimb.2023.1070271 PMC10072284

[126] S. Mauri , G. Bernardo , A. Martinez , et al., “USP8 Down‐Regulation Promotes Parkin‐Independent Mitophagy in the Drosophila Brain and in Human Neurons,” Cells 12, no. 8 (2023): 1143. 37190052 10.3390/cells12081143 PMC10136645

[127] X. Liu , M. Hebron , W. Shi , I. Lonskaya , and C. E. Moussa , “Ubiquitin Specific Protease‐13 Independently Regulates Parkin Ubiquitination and Alpha‐Synuclein Clearance in Alpha‐Synucleinopathies,” Human Molecular Genetics 28, no. 4 (2019): 548–560. 30329047 10.1093/hmg/ddy365

[128] X. Liu , K. Balaraman , C. C. Lynch , M. Hebron , C. Wolf , and C. Moussa , “Novel Ubiquitin Specific Protease‐13 Inhibitors Alleviate Neurodegenerative Pathology,” Metabolites 11, no. 9 (2021): 622. 34564439 10.3390/metabo11090622 PMC8467576

[129] L. Phu , C. M. Rose , J. S. Tea , et al., “Dynamic Regulation of Mitochondrial Import by the Ubiquitin System,” Molecular Cell 77, no. 5 (2020): 1107–1123. e1110. 32142684 10.1016/j.molcel.2020.02.012

[130] A. Y. Amerik and M. Hochstrasser , “Mechanism and Function of Deubiquitinating Enzymes,” Biochimica et Biophysica Acta (BBA)‐Molecular Cell Research 1695, no. 1–3 (2004): 189–207. 15571815 10.1016/j.bbamcr.2004.10.003

[131] F. Wang , Y. Gao , L. Zhou , et al., “USP30: Structure, Emerging Physiological Role, and Target Inhibition,” Frontiers in Pharmacology 13 (2022): 851654. 35308234 10.3389/fphar.2022.851654 PMC8927814

[132] X. Liu , M. L. Hebron , S. Mulki , et al., “Ubiquitin Specific Protease 13 Regulates Tau Accumulation and Clearance in Models of Alzheimer's Disease,” Journal of Alzheimer's Disease 72, no. 2 (2019): 425–441. 10.3233/JAD-190635 31594232

[133] A. Hyrskyluoto , C. Bruelle , S. H. Lundh , et al., “Ubiquitin‐Specific Protease‐14 Reduces Cellular Aggregates and Protects Against Mutant Huntingtin‐Induced Cell Degeneration: Involvement of the Proteasome and ER Stress‐Activated Kinase IRE1α,” Human Molecular Genetics 23, no. 22 (2014): 5928–5939. 24951540 10.1093/hmg/ddu317

[134] J. Chakraborty , S. von Stockum , E. Marchesan , et al., “USP 14 Inhibition Corrects an In Vivo Model of Impaired Mitophagy,” EMBO Molecular Medicine 10, no. 11 (2018): e9014. 30249595 10.15252/emmm.201809014 PMC6220287

[135] Z. Alexopoulou , J. Lang , R. M. Perrett , et al., “Deubiquitinase Usp8 Regulates α‐Synuclein Clearance and Modifies Its Toxicity in Lewy Body Disease,” Proceedings of the National Academy of Sciences of the United States of America 113, no. 32 (2016): E4688–E4697. 27444016 10.1073/pnas.1523597113 PMC4987833

[136] S. von Stockum , A. Sanchez‐Martinez , S. Corrà , et al., “Inhibition of the Deubiquitinase USP8 Corrects a Drosophila PINK1 Model of Mitochondria Dysfunction,” Life Science Alliance 2, no. 2 (2019): e201900392. 30988163 10.26508/lsa.201900392 PMC6467245

[137] J. R. Liang , A. Martinez , J. D. Lane , U. Mayor , M. J. Clague , and S. Urbé , “USP 30 Deubiquitylates Mitochondrial P Arkin Substrates and Restricts Apoptotic Cell Death,” EMBO Reports 16, no. 5 (2015): 618–627. 25739811 10.15252/embr.201439820 PMC4428036

[138] A. Ordureau , J. A. Paulo , J. Zhang , et al., “Global Landscape and Dynamics of Parkin and USP30‐Dependent Ubiquitylomes in iNeurons During Mitophagic Signaling,” Molecular Cell 77, no. 5 (2020): 1124–1142. e1110. 32142685 10.1016/j.molcel.2019.11.013 PMC7098486

[139] Y. Sato , K. Okatsu , Y. Saeki , et al., “Structural Basis for Specific Cleavage of Lys6‐Linked Polyubiquitin Chains by USP30,” Nature Structural & Molecular Biology 24, no. 11 (2017): 911–919. 10.1038/nsmb.3469 28945247

[140] E. Marcassa , A. Kallinos , J. Jardine , et al., “Dual Role of USP 30 in Controlling Basal Pexophagy and Mitophagy,” EMBO Reports 19, no. 7 (2018): e45595. 29895712 10.15252/embr.201745595 PMC6030704

[141] J. Yun , R. Puri , H. Yang , et al., “MUL1 Acts in Parallel to the PINK1/Parkin Pathway in Regulating Mitofusin and Compensates for Loss of PINK1/Parkin,” eLife 3 (2014): e01958. 24898855 10.7554/eLife.01958 PMC4044952

[142] S. Y.‐Y. Pang , P. W.‐L. Ho , H.‐F. Liu , et al., “The Interplay of Aging, Genetics and Environmental Factors in the Pathogenesis of Parkinson's Disease,” Translational Neurodegeneration 8 (2019): 1–11. 31428316 10.1186/s40035-019-0165-9 PMC6696688

[143] Y. Jiang , W. Bian , J. Chen , et al., “miRNA‐137‐5p Improves Spatial Memory and Cognition in Alzheimer's Mice by Targeting Ubiquitin‐Specific Peptidase 30,” Animal Models and Experimental Medicine 6, no. 6 (2023): 526–536. 38111333 10.1002/ame2.12368 PMC10757218

[144] R. D. Escarcega , K. Murambadoro , R. Valencia , et al., “Sphingosine Kinase 2 Regulates Protein Ubiquitination Networks in Neurons,” Molecular and Cellular Neuroscience 130 (2024): 103948. 38909878 10.1016/j.mcn.2024.103948 PMC13285857

[145] T. M. Durcan , M. Y. Tang , J. R. Pérusse , et al., “USP 8 Regulates Mitophagy by Removing K 6‐Linked Ubiquitin Conjugates From Parkin,” EMBO Journal 33, no. 21 (2014): 2473–2491. 25216678 10.15252/embj.201489729 PMC4283406

[146] E. F. A. Yeates and G. Tesco , “The Endosome‐Associated Deubiquitinating Enzyme USP8 Regulates BACE1 Enzyme Ubiquitination and Degradation,” Journal of Biological Chemistry 291, no. 30 (2016): 15753–15766. 27302062 10.1074/jbc.M116.718023 PMC4957057

[147] X. Liu , K. Balaraman , C. C. Lynch , et al., “Inhibition of Ubiquitin‐Specific Protease‐13 Improves Behavioral Performance in Alpha‐Synuclein Expressing Mice,” International Journal of Molecular Sciences 23, no. 15 (2022): 8131. 35897705 10.3390/ijms23158131 PMC9330474

[148] C. Banerjee , M. Roy , R. Mondal , and J. Chakraborty , “USP14 as a Therapeutic Target Against Neurodegeneration: A Rat Brain Perspective,” Frontiers in Cell and Developmental Biology 8 (2020): 727. 32850842 10.3389/fcell.2020.00727 PMC7411183

[149] K. Niu , H. Fang , Z. Chen , et al., “USP33 Deubiquitinates PRKN/Parkin and Antagonizes Its Role in Mitophagy,” Autophagy 16, no. 4 (2020): 724–734. 31432739 10.1080/15548627.2019.1656957 PMC7138199

[150] X. Chen , Q. Yang , L. Xiao , D. Tang , Q. P. Dou , and J. Liu , “Metal‐Based Proteasomal Deubiquitinase Inhibitors as Potential Anticancer Agents,” Cancer and Metastasis Reviews 36 (2017): 655–668. 29039082 10.1007/s10555-017-9701-1 PMC5721122

[151] D. Yan , X. Li , Q. Yang , et al., “Regulation of Bax‐Dependent Apoptosis by Mitochondrial Deubiquitinase USP30,” Cell Death Discovery 7, no. 1 (2021): 211. 34381024 10.1038/s41420-021-00599-6 PMC8357812

[152] T.‐S. Z. Fang , Y. Sun , A. C. Pearce , et al., “Knockout or Inhibition of USP30 Protects Dopaminergic Neurons in a Parkinson's Disease Mouse Model,” Nature Communications 14, no. 1 (2023): 7295. 10.1038/s41467-023-42876-1 PMC10643470 37957154

[153] E. V. Rusilowicz‐Jones , J. Jardine , A. Kallinos , et al., “USP30 Sets a Trigger Threshold for PINK1–PARKIN Amplification of Mitochondrial Ubiquitylation,” Life Science Alliance 3, no. 8 (2020): e202000768. 32636217 10.26508/lsa.202000768 PMC7362391

[154] J. Okarmus , J. B. Agergaard , T. C. Stummann , et al., “USP30 Inhibition Induces Mitophagy and Reduces Oxidative Stress in Parkin‐Deficient Human Neurons,” Cell Death & Disease 15, no. 1 (2024): 52. 38225227 10.1038/s41419-024-06439-6 PMC10789816

[155] A. Jones , M. Kemp , M. Stockley , K. Gibson , and G. Whitelock , “1‐Cyano‐Pyrrolidine Compounds as USP30 Inhibitors,” Mission Therapeutics (2016).

[156] E. Tsefou , A. S. Walker , E. H. Clark , et al., “Investigation of USP30 Inhibition to Enhance Parkin‐Mediated Mitophagy: Tools and Approaches,” Biochemical Journal 478, no. 23 (2021): 4099–4118. 34704599 10.1042/BCJ20210508 PMC8718267

[157] M. Kemp , M. Stockley , and A. Madin , Novel Compounds, Mission Therapeutics (2017).

[158] W. Yue , Z. Chen , H. Liu , et al., “A Small Natural Molecule Promotes Mitochondrial Fusion Through Inhibition of the Deubiquitinase USP30,” Cell Research 24, no. 4 (2014): 482–496. 24513856 10.1038/cr.2014.20 PMC3975501

[159] A. F. Kluge , B. R. Lagu , P. Maiti , et al., “Novel Highly Selective Inhibitors of Ubiquitin Specific Protease 30 (USP30) Accelerate Mitophagy,” Bioorganic & Medicinal Chemistry Letters 28, no. 15 (2018): 2655–2659. 29935771 10.1016/j.bmcl.2018.05.013

[160] H. Luo , J. Krigman , R. Zhang , M. Yang , and N. Sun , “Pharmacological Inhibition of USP30 Activates Tissue‐Specific Mitophagy,” Acta Physiologica 232, no. 3 (2021): e13666. 33890401 10.1111/apha.13666 PMC8266733

[161] D. P. O'Brien , H. B. Jones , F. Guenther , et al., “Structural Premise of Selective Deubiquitinase USP30 Inhibition by Small‐Molecule Benzosulfonamides,” Molecular & Cellular Proteomics 22, no. 8 (2023): 100609. 37385347 10.1016/j.mcpro.2023.100609 PMC10400906

[162] E. V. Rusilowicz‐Jones , F. G. Barone , F. M. Lopes , et al., “Benchmarking a Highly Selective USP30 Inhibitor for Enhancement of Mitophagy and Pexophagy,” Life Science Alliance 5, no. 2 (2022): e202101287. 34844982 10.26508/lsa.202101287 PMC8645336

[163] X. Qin , R. Wang , H. Xu , et al., “Identification of an Autoinhibitory, Mitophagy‐Inducing Peptide Derived From the Transmembrane Domain of USP30,” Autophagy 18, no. 9 (2022): 2178–2197. 34989313 10.1080/15548627.2021.2022360 PMC9397470

[164] B.‐H. Lee , M. J. Lee , S. Park , et al., “Enhancement of Proteasome Activity by a Small‐Molecule Inhibitor of USP14,” Nature 467, no. 7312 (2010): 179–184. 20829789 10.1038/nature09299 PMC2939003

[165] M. Boselli , B.‐H. Lee , J. Robert , et al., “An Inhibitor of the Proteasomal Deubiquitinating Enzyme USP14 Induces Tau Elimination in Cultured Neurons,” Journal of Biological Chemistry 292, no. 47 (2017): 19209–19225. 28972160 10.1074/jbc.M117.815126 PMC5702663

